# Epigenetic regulation of ferroptosis via ETS1/miR-23a-3p/ACSL4 axis mediates sorafenib resistance in human hepatocellular carcinoma

**DOI:** 10.1186/s13046-021-02208-x

**Published:** 2022-01-03

**Authors:** Yuanjun Lu, Yau-Tuen Chan, Hor-Yue Tan, Cheng Zhang, Wei Guo, Yu Xu, Rakesh Sharma, Zhe-Sheng Chen, Yi-Chao Zheng, Ning Wang, Yibin Feng

**Affiliations:** 1 School of Chinese Medicine, The University of Hong Kong, Hong Kong, China; 2 Centre for Chinese Herbal Medicine Drug Development, School of Chinese Medicine, Hong Kong Baptist University, Hong Kong, China; 3 Department of Pharmacy, Union Hospital, Tongji Medical College, Huazhong University of Science and Technology, Wuhan, Hubei Province People’s Republic of China; 4 Proteomics and Metabolomics Core Facility, The University of Hong Kong, Hong Kong, China; 5 Department of Pharmaceutical Sciences, College of Pharmacy and Health Sciences, St. John’s University, Queens, NY USA; 6 School of Pharmaceutical Sciences, Zhengzhou University, Zhengzhou, Henan Province People’s Republic of China

**Keywords:** MiR-23a-3p, Sorafenib resistance, ETS1, Ferroptosis, ACSL4, Hepatocellular carcinoma

## Abstract

**Background:**

Drug resistance to sorafenib greatly limited the benefits of treatment in patients with hepatocellular carcinoma (HCC). MicroRNAs (miRNAs) participate in the development of drug resistance. The key miRNA regulators related to the clinical outcome of sorafenib treatment and their molecular mechanisms remain to be identified.

**Methods:**

The clinical significance of miRNA-related epigenetic changes in sorafenib-resistant HCC was evaluated by analyzing publicly available databases and in-house human HCC tissues. The biological functions of miR-23a-3p were investigated both in vitro and in vivo. Proteomics and bioinformatics analyses were conducted to identify the mechanisms that regulating miR-23a-3p. Luciferase reporter assay and chromatin immunoprecipitation (ChIP) assay were used to validate the binding relationship of miR-23a-3p and its targets.

**Results:**

We found that miR-23a-3p was the most prominent miRNA in HCC, which was overexpressed in sorafenib non-responders and indicated poor survival and HCC relapse. Sorafenib-resistant cells exhibited increased miR-23a-3p transcription in an ETS Proto-Oncogene 1 (ETS1)-dependent manner. CRISPR-Cas9 knockout of miR-23a-3p improved sorafenib response in HCC cells as well as orthotopic HCC tumours. Proteomics analysis suggested that sorafenib-induced ferroptosis was the key pathway suppressed by miR-23a-3p with reduced cellular iron accumulation and lipid peroxidation. MiR-23a-3p directly targeted the 3′-untranslated regions (UTR) of ACSL4, the key positive regulator of ferroptosis. The miR-23a-3p inhibitor rescued ACSL4 expression and induced ferrotoptic cell death in sorafenib-treated HCC cells. The co-delivery of ACSL4 siRNA and miR-23a-3p inhibitor abolished sorafenib response.

**Conclusion:**

Our study demonstrates that ETS1/miR-23a-3p/ACSL4 axis contributes to sorafenib resistance in HCC through regulating ferroptosis. Our findings suggest that miR-23a-3p could be a potential target to improve sorafenib responsiveness in HCC patients.

**Supplementary Information:**

The online version contains supplementary material available at 10.1186/s13046-021-02208-x.

## Background

Hepatocellular carcinoma (HCC) accounts for 75% ~ 85% of primary liver cancers and its incidence is rising all over the world, especially in Asia [1]. The mortality of HCC ranks third globally according to the GLOBOCAN 2020 (http://gco.iarc.fr/) and is expected to increase by 60.9% in 2040. Due to the difficulty in early diagnosis, most patients with HCC are diagnosed at late stages with limited treatment choices. Therefore, systemic therapy is the only therapeutic option [2]. Sorafenib is the first drug approved by the Food and Drug Administration (FDA) as the first-line systemic treatment for advanced HCC. Sorafenib functions as a multiple-targeted tyrosine kinase inhibitor (TKI) [3]. Although the Sorafenib HCC Assessment Randomized Protocol (SHARP) trial has shown promising outcomes for patients with advanced HCC, with approximately 3 months extension in median overall survival, only a very limited number of patients could benefit from sorafenib treatment due to the development of drug resistance within 6 months [4]. The efficacy and effectiveness of sorafenib treatment are greatly challenged by the inherent and acquired drug resistance [5]. Therefore, identifying the key issues involved in sorafenib resistance is critical for effective management of HCC patients.

Currently, multiple mechanisms have been demonstrated to confer sorafenib resistance in HCC, including the expression of the cell membrane transporter proteins that mediate drug uptake and efflux, alteration in molecular targets and related signalling pathways, tumour heterogeneity and plasticity, resistance to cell death and epigenetic modifications, and others [6, 7]. Ferroptosis is a newly identified iron-dependent regulated cell death (RCD) characterized by the accumulation of lipid reactive oxygen species (ROS). Increasing evidence revealed that sorafenib induces ferroptosis, leading to iron toxicity and lipid peroxidation in various cancers [8]. Therefore, ferroptosis regulators were considered to be valuable targets for enhancing sorafenib response.

Among the epigenetic modifications on sorafenib resistance, miRNAs have been regarded as a group of master regulators participating in multiple cellular processes in HCC [9]. For example, the upregulation of miR-222, miR-378a, miR-494 and miR-93 was observed in sorafenib resistant HCC and these miRNAs were found to target the PTEN/Akt/mTOR signalling pathway [10–13]; the downregulation of miR-let-7, miR-142-3p, miR-34a and miR-541 suppresses HCC cell death via targeting genes associated with autophagy and apoptosis [14–17]. Dysregulated miR-122, miR-125-5p, miR-181a and miR-486-3p could alter the activity of tyrosine kinases and result in blunted sorafenib response [18–21]. However, few miRNAs were reported to affect ferroptosis in the development of sorafenib resistance in HCC.

In the present study, we observed that the upregulation of miR-23a-3p was responsible for the acquisition of sorafenib resistance. MiR-23a-3p acted as a direct suppressor of ferroptosis by targeting the 3’UTR of ACSL4. The ETS1 was identified as the upstream transcription factor (TF) of miR-23a-3p and was activated following sorafenib treatment. Therefore, targeting miR-23a-3p may sensitize HCC response to sorafenib treatment.

## Materials and methods

### Weighted gene co-expression network construction and identification of clinically significant modules

The co-expression network of miRNAs from the GSE56059 was constructed using the WGCNA package in the R software. The sample clustering was plotted to eliminate the outliers. We selected β = 7 as the appropriate soft-thresholding power and ensure scale-free topology, R2 > 0.9. The adjacency was transformed to TOM with TOM similarity and its dissimilarity (dissTOM). Using the dynamic tree cut method, we set at least 30 co-expressed miRNAs to be aggregated in each module eigengenes. The correlation between clinical characteristics and module eigengenes (ME) was quantified with R and certain *P*-values were obtained. By using the gene significance (GS) and module membership (MM) measures, the relationship between intramodular genes that have high significance to the clinical information and MM was confirmed.

### Human samples

A tissue microarray chip containing 90 pairs of human HCC samples matched to their adjacent normal liver tissues and the associated clinicopathological information was purchased from Shanghai OUTDO Biotech Co., Ltd., Shanghai, China (LivH180Su08).

### Double in situ hybridization (ISH)

The protocol of double ISH was based on the previous publication [22] with some modifications. Briefly, the general rehydration steps were followed by 20-min digestion with 5 μL/mL proteinase K (Sigma P8044) at 37 °C. Samples were pre-hybridized in solution at 83 °C for 30 min. MiR-23a-3p probe (Qiagen 339,111) was denatured at 65 °C for 5 min and immediately chilled on ice for 5 min. Then the hybridization was performed in solution with 40 nM of miR-23a-3p probe at 53 °C overnight. The slide was washed stringently with 1x saline-sodium citrate buffer (SSC) at 53 °C for 10 min twice and 0.5x SSC at room temperature for 10 min. The staining steps followed the manual of Alexa Fluor™ 488 tyramide kit (Thermo Fisher B40932). It was processed with standard immunofluorescence protocol using the 594/555 secondary antibody and captured by LSM 780 confocal microscope (Carl Zeiss).

### Animal experiment

All animal protocols were approved by the Committee on the Use of Live Animals in Teaching and Research of the University of Hong Kong.

#### In vivo generation of sorafenib resistant HCC

Parental MHCC97L cells (2 × 10^6^ cells/mouse) were subcutaneously injected into the 4-to-5-week-old NOD-SCID mice. When the tumours reached a volume of around 50–100 mm^3^ (calculated by the formula 4/3π(D/2)(d/2)^2^, where D and d represent the minor and major axis of the tumour, respectively), the maximum tolerated dose of sorafenib (50 mg/kg) was given to the mice by oral gavage daily until the drug resistance occurred, denoted as the drug resistant group. For the control, the wild type group was treated with the vehicle (0.5% CMC-Na). The tumour size and body weight were measured every 3 days. The isolation of tumour cells followed the method as previously described [23].

#### Orthotopic implantation of HCC in mice

The 1 × 10^6^ luciferase-tagged MHCC97L cells were subcutaneously injected into the right flank of the NOD-SCID mouse. Once the tumour diameter reached 10 mm by calliper measurement, the mice were sacrificed. The tumour was harvested and cut into small cubes (~ 1 mm^3^). One tumour cube was implanted to the left lobe of the liver of a 5-week-old BALB/cAnN-nu mouse. Tumour growth in the orthotopic HCC model was monitored by obtaining the bioluminescence signal with the IVIS Spectrum system (PerkinElmer) weekly.

### Cell culture, reagents and plasmids

#### Cells

The PLC/PRF/5 cell line was purchased from the American Type Culture Collection (ATCC) (VA, USA). MHCC97L with luciferase tag was a gift from Prof. Man Kwan from the Department of Surgery, the University of Hong Kong. 293FT was a gift from Prof. Xinyuan Guan from the Department of Clinical Oncology, the University of Hong Kong. The PLC/PRF/5 and MHCC97L cells were cultured in DMEM, high glucose (Gibco) with 10% fetal bovine serum and supplemented with 1% penicillin/streptomycin. 293FT cells were cultured in the complete DMEM, high glucose medium with 1 mM sodium pyruvate.

#### Reagents

Sorafenib (S-8502) was purchased from LC laboratories. Ferrostatin-1 was purchased from MeChemExpress. Lipofectamine 3000 reagent (Invitrogen) for transient transfection was used according to the manufacturer’s instructions.

#### Plasmids, miRNA mimics and RNA interference

The miR-23a-3p mimics and NC, Anti-miR-23a-3p and Anti-NC, ETS-1 siRNAs, ACSL4 siRNAs were commercially obtained from GenePharma, China. The plasmids pLenti-III-miR-Off (23a-KO) and its control (Scramble), and ACSL4 3’UTR luciferase reporter vector were purchased from Applied Biological Materials (Canada). Lentivirus packaging vectors: pRSV-Rev, pMDLg/pRRE were gifts from Didier Trono (Addgene plasmid #12251 and #12253; http://n2t.net/addgene:12253; RRID: Addgene_12,253) [24]. Lentivirus enveloping plasmid pCMV-VSV-G was a gift from Bob Weinberg (Addgene plasmid #8454; http://n2t.net/addgene:8454; RRID: Addgene_8454) [25]. pGL3-23P639 luciferase reporter vector was a gift from Narry Kim (Addgene plasmids #51388; http://n2t.net/addgene:51388; RRID: Addgene_51,388) [26].

### Mature miRNA and Pri-miRNA assays

#### Mature miRNA assay

The total RNA was subjected to reverse transcription with miCURY LNA RT kit (Qiagen) and the quantitative real-time PCR (qPCR) was conducted on LightCycler 480 (Roche) with miRCURY LNA miRNA PCR Assay (Qiagen). The primer sets of miR-23a-3p and its control U6 were purchased from Qiagen. Relative miR-23a-3p expression was normalized to U6 snRNA expression level. The comparative Ct method was used for data analysis, and all experiments were performed in triplicates.

#### Pri-miRNA assay

The pri-miR-23a detection was conducted as described previously [27] by using TaqMan primary microRNA assay kit (Applied Biosystems) on the LightCycler 480 (Roche). The specific primer sets for pri-miR-23a and its control *GAPDH* were listed in Supplementary Table S2.

##### Quantitative real-time PCR (qPCR)

Total RNA was isolated using RNAiso Plus reagent (Takara). Reverse transcription was performed using a HiScript III First Strand cDNA Synthesis Kit (Vazyme, China) by following the manufacturer’s protocol. RT-qPCR assay was performed with SYBR green PCR master mix reagent (Vazyme, China) on the LightCycler 480 (Roche). The primer sets were listed in Supplementary Table S2.

##### Establishment of 23a-KO MHCC97L cell lines

Lentivirus packaging vectors (pRSV-Rev and pMDLg/pRRE), an enveloping vector (pCMV-VSV-G), and 23a-KO/Scramble at the ratio of 1:1:1:2 were co-transfected to 293FT cells with Lipofectamine 3000. The culture medium of 293FT was collected after 48 h and 72 h, filtered with a 0.45 μm filter and applied to transduce MHCC97L. Stable cell lines were selected with 0.3 μg/ml puromycin for 7 days. The knockout efficiency was determined by RT-qPCR.

##### Cell viability assay

Cells seeded onto a 96-well plate (0.5 × 10^4^ cells/well) were transfected with 10 nM miR-23a-3p mimics or 30 nM Anti-miR-23a for 48 h and treated with different doses of sorafenib for another 24 h. Cell viability was determined by MTT assay. The 10 μL of 0.5 mg/mL MTT was added to the well for 4-h incubation. The medium was then removed, and the residue was dissolved in DMSO. The light absorbance at 490 nm was measured by Multiskan MS microplate reader (Labsystems, Finland).

##### Proteomics

A total of 2 × 10^6^ MHCC97L cells were transfected with 10 nM miR-23a-3p mimics (AUCACAUUGCCAGGGAUUUCC) and its control NC (UUCUCCGAACGUGUCACGUTT) for 48 h and subjected to 15 μM sorafenib treatment for another 24 h. The cells were harvested by centrifugation at 300 g after trypsinization. Sample processing and label-free LC-MS/MS were performed by Proteomics and Metabolomics Core Facility, LKS Faculty of Medicine, The University of Hong Kong.

##### Chromatin immunoprecipitation (ChIP)-qPCR

ChIP was performed by following the protocol of the EZ-Magna ChIP A/G Chromatin Immunoprecipitation kit (Sigma-Aldrich). One 10 cm-dish of MHCC97L (1 × 10^7^) treated with sorafenib or vehicle for 48 h was fixed with 1% paraformaldehyde (PFA) and proceeded to washing steps with cold PBS. Then the cells were harvested and subjected to cellular and nuclear lysis. The whole nuclear lysate was sheared by a sonicator with optimal condition (7 s pulse on, 10 s pulse off, 15 cycles, 40% amplitude) to yield 200–700 bp DNA. Five microlitre of sheared lysate was aliquoted as Input. Fifty microlitre of the sheared lysate (the equivalent of 1 × 10^6^ cells) was subjected to immunoprecipitation by overnight incubation of either anti-ETS-1 antibody (Cell Signaling Technology, 14,069) or IgG control. The immunoprecipitated DNA and Input DNA were purified and amplified by qPCR with primers listed in Supplementary Table S2.

##### Luciferase reporter assay

For miR-23a promoter activity, pGL3-23P639 luciferase reporter vector containing pri-miR-23a promoter (ranges from − 603 to + 36 nt) together with *Renilla* luciferase reporter vector was transfected at the ratio of 25:1 to 293FT cells. Meanwhile, either NC or siETS1 was co-transfected with luciferase reporter vectors to 293FT cells. For ACSL4 3’UTR luciferase activity, ACSL4–3’UTR luciferase reporter vector and *Renilla* luciferase reporter vector co-transfected with either miR-23a-3p mimics or Anti-miR-23a to 293FT cells for 48 h. After 48 h treatment, cells were lysed and detected by the Dual-Luciferase Reporter Assay System (Promega) with a luminometer. Luciferase activity was represented by a ratio of firefly: *Renilla* luminescence.

### Flow cytometry analysis

#### Cell death determination

Pharmingen FITC Annexin V Apoptosis Detection Kit (BD Biosciences) was used to detect cell apoptosis following the manufacturer’s protocol. Cells were seeded in a 6-well plate at a concentration of 2.5 × 10^5^ cells/well and transfected with 10 nM miR-23a-3p or 30 nM anti-miR-23a-3p. After transfection of 48 h, sorafenib (15 μM for MHCC97L, 11 μM for PLC/PRF/5) was added and incubated for 24 h. All cells, including the floating cells in the culture medium, were collected and stained with 5 μL FITC Annexin V and 5 μL PI for 15 min at room temperature in the dark. Then the samples were subjected to flow cytometric analysis (BD FACSCanto-II Analyzer).

#### Intracellular chelatable iron determination

Cells after treatment were washed twice with warm (37 °C) HBSS and incubated with 20 μM of Phen Green SK (PGSK) diacetate (ThermoFisher, P14313) as intracellular chelatable iron indicator for 15 min in HBSS. The chelation of iron by PGSK could lead to a dynamic fluorescence quenching. The fluorescence was detected by flow cytometric analysis (NovoCyte Advanteon) within 30 min.

#### Reactive Oxygen Species (ROS) measurement

The intracellular ROS level was measured by 2′,7′-Dichlorofluorescin diacetate (Sigma-Aldrich). In brief, cells after treatment were stained with 5 μM of 2′,7′-Dichlorofluorescin diacetate in PBS for 5 min and subjected to flow cytometric analysis (NovoCyte Advanteon) within 15 min. All data were analysed by the FlowJo 7.6.1 software.

##### Lipid peroxidation determination

Cells were seeded onto sterilized coverslips and treated accordingly with added 10 μM BODIPY 493/503 (ThermoFisher) for 30 min. Cells were then fixed with 4% PFA for 15 min and stained with DAPI for 5 min after washing with PBS. Images were captured with LSM 780 confocal microscope (Carl Zeiss, Germany).

##### Immunofluorescence staining

The 5 μm thick paraffin-embedded sections were subjected to dewaxing and rehydration according to standard procedures. The antigen retrieval was conducted with 10 mM citrate buffer (Sigma-Aldrich, USA), followed by blocking with 10% goat serum for 1 h. Sections were incubated with anti-cleaved caspase-3 (Cell Signaling Technology, 9664) at 4 °C overnight and washed in PBS. Then sections were counterstained with secondary antibody Alexa Fluor 568 (Invitrogen, A-11031) for 1 h and washed in PBS. DAPI (Invitrogen, D1306) staining was performed for 5 min and washed in PBS. Afterwards, sections were mounted with fluorescence mounting medium (Dako, Denmark). Sections after primary antibody incubation should be protected from light. Images were captured with LSM 780 confocal microscope. Cleaved caspase-3-positive cells were apoptotic cells.

##### Immunoblotting

Cell pellets were lysed with RIPA buffer supplemented with proteinase inhibitors and phosphatase inhibitors on ice for 30 min and centrifugated at 12,000 rpm at 4 °C for 10 min. Total protein concentration was measured by Bradford assay, and equal amounts of protein were resolved by SDS-PAGE and transferred onto the PVDF membrane. The membrane was blocked with 5% BSA in TBST and then incubated with primary antibodies at 4 °C overnight. An appropriate secondary antibody was incubated at room temperature for 2 h. The immunoreactivities were detected by the Amersham ECL Prime Western Blotting Detection Reagent (Cytiva) and captured by a chemiluminescence imaging system (Bio-Rad). The following primary antibodies were commercially obtained: anti-PARP (#9532), anti-caspase-3 (#9662), anti-cleaved-caspase-3 (#9661), anti-ETS-1 (#14069), anti-ACTIN (#4970), anti-GPX4 (#52455), anti-p-AKT (#4060), anti-AKT (#4691), anti-ERK (#4695), and anti-p-ERK (#8544) from Cell Signaling Technology; anti-SLC7A11 (A2413) and anti-ACSL4 (A16848) from Abclonal.

#### Statistical analysis

All statistical analyses were performed by GraphPad Prism 7 software (CA, USA). Two-tailed Student’s t-test and Mann-Whitney U test were used for two-group comparisons with normal distribution and non-normal distribution, respectively. Kruskal-Wallis test and one-way ANOVA with Tukey’s multiple comparison test were used for multi-group comparisons with non-parametric and parametric test, respectively. *P*-value less than 0.05 was considered to be statistically significant.

## Results

### MiR-23a-3p predicted poor sorafenib response and HCC relapse in HCC patients

**Fig. 1:**
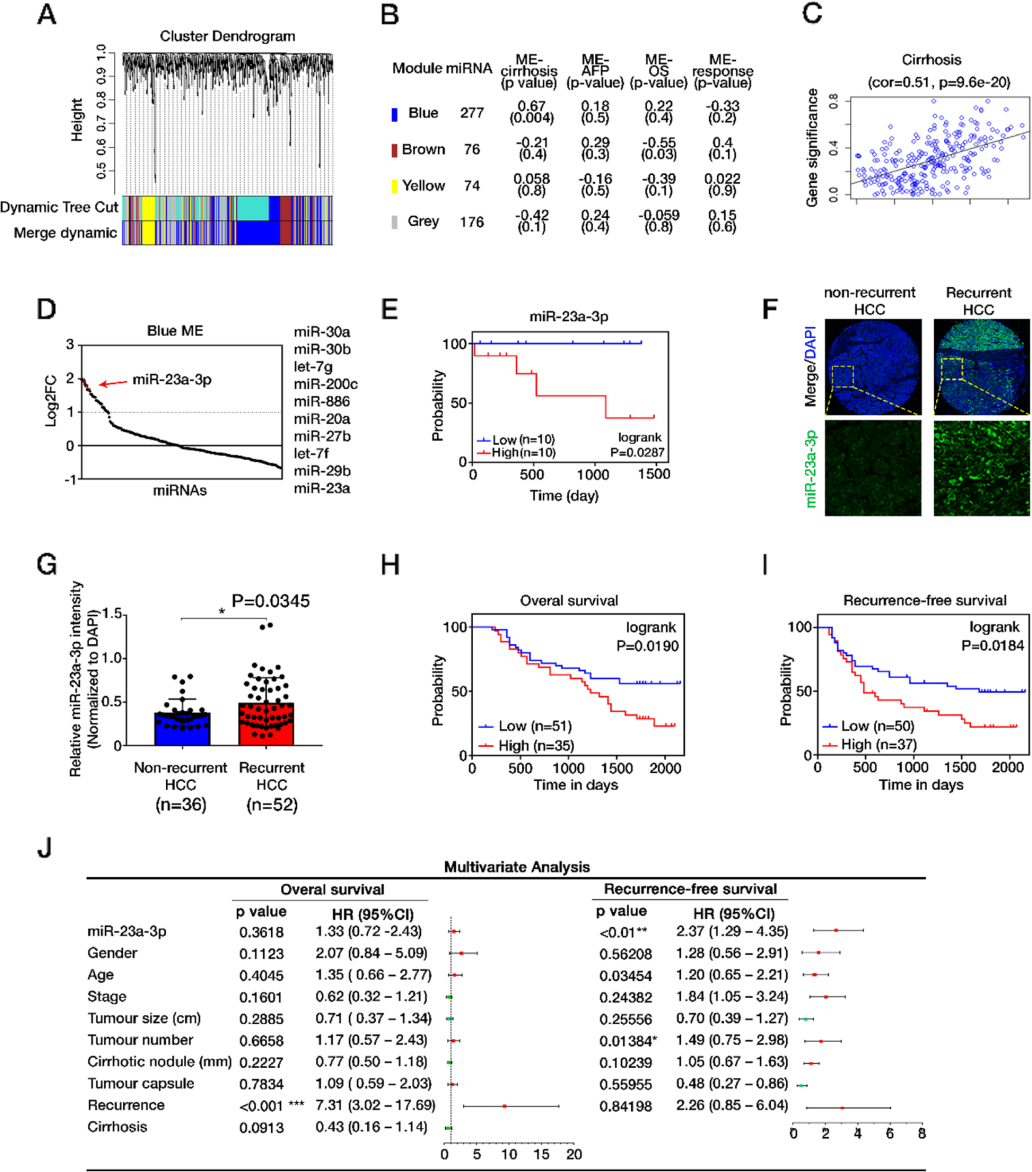
The evaluation of clinical importance of miR-23a-3p. **A** Dendrogram of miRNAs clustered based on a dissimilarity measure (1-TOM) through WGCNA analysis. **B** Module member count, ME-clinical traits correlation (Pearson) and *P*-value indicated for each module. **C** Scatterplots showing the correlations between gene module membership in the blue module and gene significance for cirrhosis. **D** A dot plot showing the ranking of miRNAs according to their log2 fold change. **E** Progression-free survival curve of miR-23a-3p in HCC patients treated with sorafenib. Data were gathered from the GSE56059. **F** Representative images of miR-23a-3p and DAPI staining in the human HCC tissue microarray. **G** Increased miR-23a-3p expression observed in recurrent HCC, *P* = 0.0345. **H** OS and **I** RFS outcomes based on Kaplan-Meier survival analysis. The increased expression of miR-23a-3p was significantly associated with poor OS and DFS with *P*-values 0.0190 and 0.0184, respectively. **F** Multivariate Cox analysis showing the association between clinicopathologic factors and HCC patient survival outcome. The expression of MiR-23a-3p is significant only in RFS. **P < 0.01, *P < 0.05

To identify dysregulated miRNAs associated with sorafenib resistance in HCC, we retrieved the miRNA expression profile from the GSE56059 in the GEO database. This dataset included HCC biopsies from patients administered with sorafenib and defined patients with progressive disease (pd) as non-responders, and others as responders to sorafenib. MiRNAs were analysed through WGCNA with soft-thresholding power (β) 7 (sample clustering and analysis of the scale-free fit index for β can be found in Fig. S1A, B). A total of four merged modules were identified by gathering co-expressed miRNAs, including blue [277], brown [76], yellow [74] and grey modules[176] (Fig. 1A). When associated ME with clinical traits in each module, blue ME showed a significant correlation with cirrhosis in HCC and a negative correlation with response to sorafenib. Therefore it was submitted to further analysis (Fig. 1B, C, Fig. S1C). The top 10 upregulated miRNAs are miR-30a, miR-30b, let-7 g, miR-200c, miR-886, miR-20a, miR-27b, let-7f, miR-29b, and miR-23a (Fig. 1D). The overall survival analysis on patients with liver cancer retrieved from the Kaplan-Meier Plotter indicated that higher miR-23a prominently contributed to worse survival of HCC (AUROC = 0.6101, *P* = 0.0052) (Fig. S1D). Moreover, patients with overexpression of miR-23a-3p showed inferior progression-free survival (PFS) with sorafenib treatment, suggesting the negative correlation of miR-23a-3p with patients response to sorafenib (Fig. 1E).

We also evaluated the clinical significance of miR-23a-3p in HCC by in situ hybridization of miR-23a-3p on a human HCC tissue microarray (TMA) containing 90 pairs of HCC specimens and their normal adjacent liver tissues (NATs). There was no significant difference in miR-23a-3p expression between HCC, NATs and different grades of HCC tissues (Fig. S1E, F). We observed higher expression of miR-23a-3p in patients with recurrent HCC (Fig. 1F, G). To uncover the association between clinicopathologic factors and HCC patient survival outcomes, we employed both univariate and multivariate analysis using the Cox regression survival model. At univariate analysis, poor overall survival (OS) and recurrence-free survival (RFS) were associated with overexpression of miR-23a-3p (Fig. 1H, I, Fig. S1G). On the other hand, miR-23a-3p expression was significantly associated with RFS [hazard ratio (HR) = 2.37, *P* = 0.006] at the multivariate analysis, indicating that miR-23a-3p was an independent risk factor of HCC relapse (Fig. 1J).

### Upregulation of miR-23a-3p was responsible for the acquisition of sorafenib resistance in HCC

**Fig. 2:**
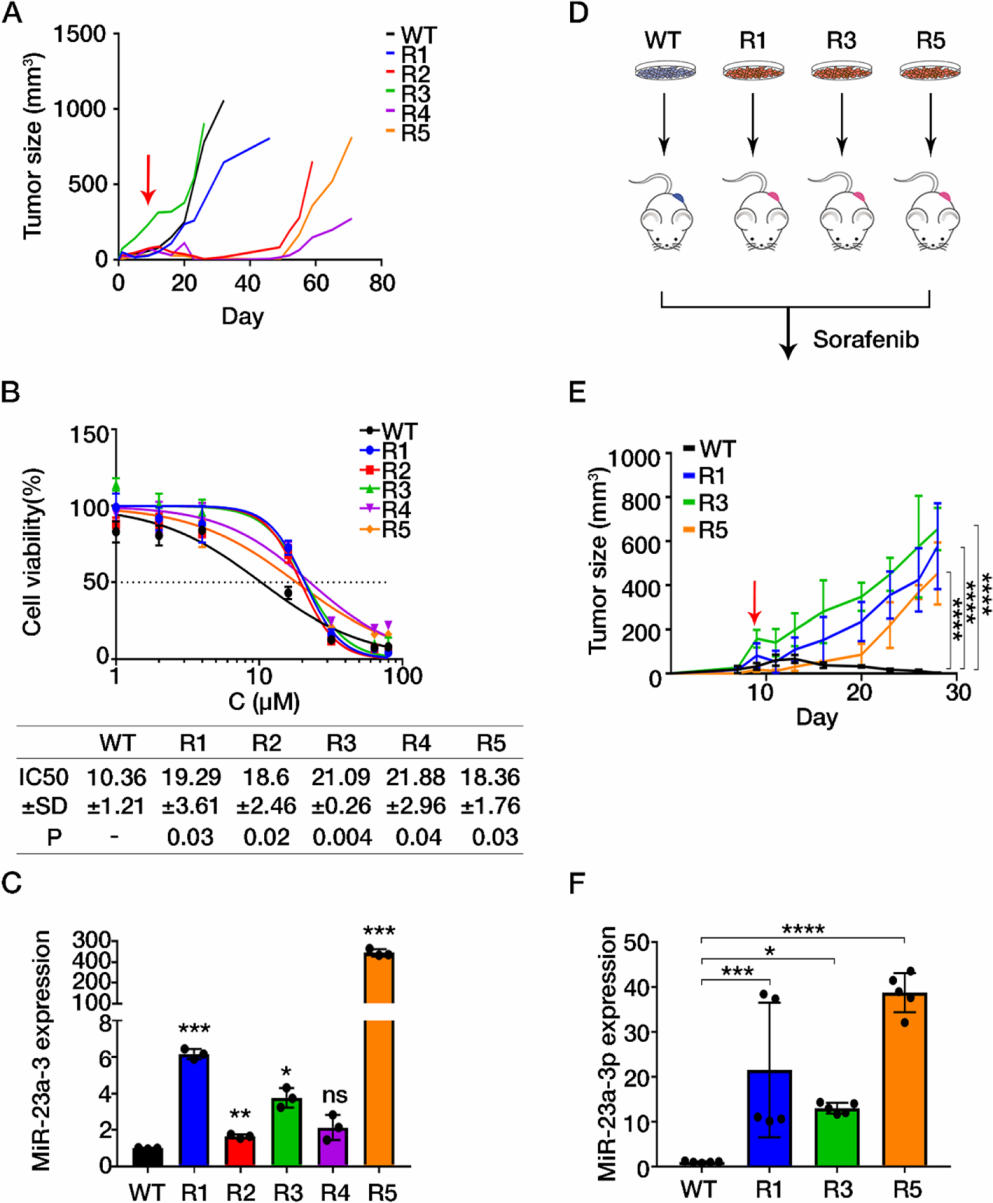
Upregulated miR-23a-3p in in vivo-generated sorafenib resistant HCC cells. **A** Tumour growth of mice was recorded every 3 days. WT: vehicle group; R1–5: sorafenib-treated group. **B** Data are represented as the percentage of WT and R1-R5 cells, and each of the experiment was performed in triplicate. The IC_50_ values of sorafenib in tumour cells for 24 h were determined by MTT assay and were calculated by GraphPad Prism 7 with the equation of Y=Bottom+(Top-Bottom)/(1 + 10^((LogIC_50_X)*HillSlope)). Mean ± SD of IC_50_ was displayed and P-value of comparison between WT and sorafenib resistance group was shown. **C** The expression of miR-23a-3p in both parental and in vivo-generated sorafenib resistant cell lines. **D** and **E** Tumour growth in the re-injected mouse models. WT: mice with parental cells; R1/3/5: mice with in vivo-generated sorafenib resistant cells. **F** The expression of miR-23a-3p in the re-injected mouse model. The arrow indicates the start of sorafenib administration. One-way ANOVA, *P < 0.05, **P < 0.01, ***P < 0.005, ****P < 0.0001

As upregulated miR-23a-3p was observed in HCC patients with poor sorafenib response, whether overexpression of miR-23a-3p was related to the development of sorafenib resistance deserves further clarification. A previous study showed that both intrinsic and extrinsic mechanisms could trigger drug resistance [28]. We therefore established an in vivo-generated sorafenib-resistant HCC cell line. In brief, HCC MHCC97L cells were subcutaneously injected into the right flank of NOD/SCID immunodeficient mice. Upon tumour growth, mice were orally administrated with sorafenib or vehicle daily. Mice in the vehicle group (WT) presented continuous tumour growth. However, mice upon sorafenib treatment (R1–5) presented a temporary reduction in size with rapid regrowth after long exposure to sorafenib, indicating the acquisition of sorafenib resistance (Fig. 2A). Their body weight gradually recovered from the temporary loss at the beginning under sorafenib treatment also suggested the adaptation towards sorafenib treatment (Fig. S2A). To detect their response to sorafenib, we isolated HCC cells from the tumours and measured the half-maximal inhibitory concentration (IC_50_) of sorafenib via MTT assay. Those cells from the regrown tumours (R1–5) exhibited a higher IC_50_ value of sorafenib, indicating the characteristic of sorafenib resistance (Fig. 2B). Relatively high level of miR-23a-3p was observed in those in vivo-generated sorafenib resistant cells (R1–5) (Fig. 2C). To determine whether the sorafenib resistant characteristics could be maintained after several passages, we subcutaneously re-injected three resistant lines (R1, R3 and R5) and parental cells respectively into NOD/SCID mice (*n* = 5) as shown in Fig. 2D. Tumours of resistant lines showed no responses to sorafenib treatment (Fig. 2E, body weight of mice can be found in Fig. S2B), suggesting the acquired sorafenib resistance in these cell lines could be maintained across passages. The expression of miR-23a-3p in sorafenib resistant cells was augmented over tenfold compared to the parental tumours (Fig. 2F). These results suggested that the increased expression of miR-23a-3p in both patients biopsies and in vivo-generated sorafenib resistant cells might be responsible for the acquisition of sorafenib resistance in HCC.

### MiR-23a-3p upregulation by sorafenib was directly stimulated by ETS1

**Fig. 3:**
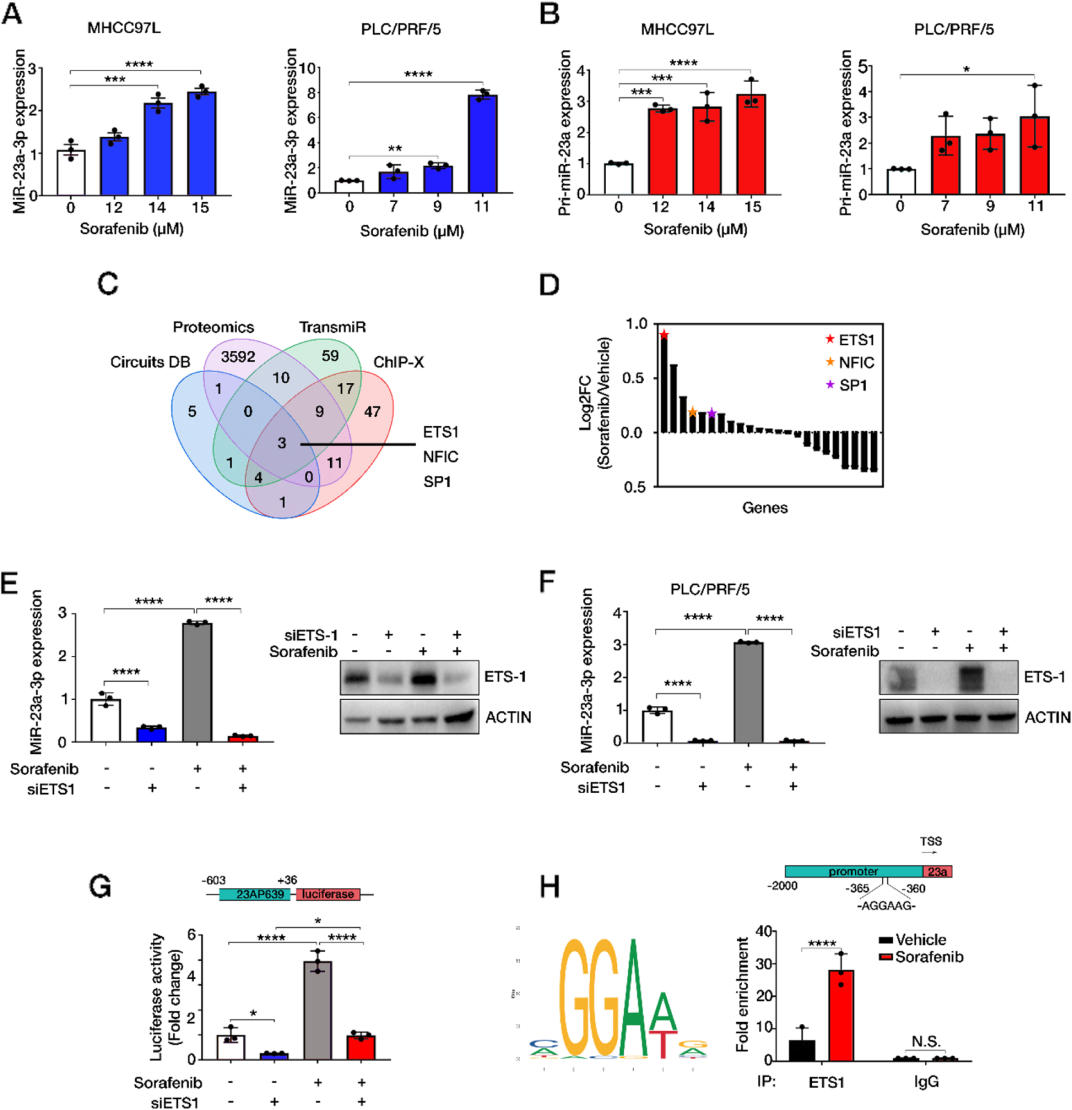
ETS1 directly stimulates miR-23a-3p transcription upon sorafenib treatment. **A** The expression of miR-23a-3p and **B** pri-miR-23a in MHCC97L and PLC/PRF/5 cells treated with sorafenib for 24 h. The doses of sorafenib were corresponding to 0, IC_10_, IC_15_, and IC_20_ values of sorafenib. **C** Venn diagram showing the intersections of data from proteomics analysis, ChIP-X, TransmiR and Circuit. Three TFs: ETS1, NFIC, and SP1 were the common TFs. **D** Gene ranking of potential TFs of proteomics data according to their log2FC. ETS1 was the highest upregulated TF. **E** The expression of miR-23a-3p in MHCC97L and **F** PLC/PRF/5 by qRT-PCR and the expression of ETS1 by immunoblotting. **G** Luciferase activity of pGL-23AP639 in HEK293. One-way ANOVA, *p* < 0.005***, *p* < 0.0001****. **H** The binding motif of ETS-1 on miR-23a promoter and the fold enrichment of fragments of miR-23a promoter was higher in sorafenib treated MHCC97L after the pulldown by ETS-1 Ab. The IgG Ab group was set as the negative control. Unpair t-test, *p* < 0.0001****

To identify whether the transcription of miR-23a-3p could be directly regulated by sorafenib, we treated two commonly used HCC cell lines, MHCC97L and PLC/PRF/5 with sorafenib. The IC_50_ values of sorafenib were calculated by plotting cell viability versus drug concentration (Fig. S3A). The downregulation of phosphorylated extracellular signal-regulated kinase (ERK) indicated positive responses to sorafenib in HCC cell lines (Fig. S3B). At doses far lower than the IC_50_ value of sorafenib, mature miR-23a-3p was induced in a dose-dependent manner (Fig. 3A). Its primary form (pri-miR-23a) was also increased by sorafenib (Fig. 3B), indicating that sorafenib treatment stimulated the transcription activity of miR-23a-3p.

So far, several transcription factors (TFs) associated with miR-23a-3p expression have been reported to facilitate miR-23a-associated regulations during cancer development [29–31]. However, the key TF activated by sorafenib that regulates miR-23a-3p transcription remained unidentified. Proteomics analysis was performed on MHCC97L cells treated with and without sorafenib. A total of 3626 proteins in proteomics data were inputted to Enrichr using gene set library: ENCODE and ChEA consensus TFs from ChIP-X (https://genome.ucsc.edu/ENCODE/) and 23 proteins were enriched as potential TFs. We integrated these 92 TFs with predicted TFs of miR-23a-3p in TransmiR [103] and CircuitDB [15] and obtained 3 overlapped TFs: ETS1, NFIC, SP1 (Fig. 3C). As the ETS Proto-Oncogene 1 (ETS1) was the highest-ranking TF in the proteomics data, thereby it was subjected to further validation (Fig. 3D).

It was well known that ETS1 is a member of the ETS family, which presents a conserved ETS DNA-binding domain recognizing GGAA/T sequence in target genes. High level of ETS-1 was reported to be associated with poor prognosis of patients with advanced HCC who were treated with sorafenib. High level of ETS-1 promoted sorafenib resistance by inducing the expression of multidrug resistance-related genes [32]. To investigate whether ETS1 was responsible for miR-23a-3p overexpression, we used ETS1 siRNA to inhibit ETS1 expression (Fig. S3C). It was found that inhibiting ETS1 reduced miR-23a-3p expression and effectively restrained the sorafenib-induced enhancement of miR-23a-3p in both MHCC97L and PLC/PRF/5 cells (Fig. 3E, F). To confirm the transcriptional regulation of ETS-1 on miR-23a-3p promoter, we detected the luciferase activity of the miR-23a-3p promoter. It was revealed that ETS1 inhibition potently neutralized sorafenib-induced transcriptional activation, which is consistent with the regulation on miR-23a-3p expression (Fig. 3G). Prediction for the binding motif of ETS1 in JASPER suggested that AGGAAG from − 360 to -365 nt before miR-23a-3p promoter was one of the key motifs. To experimentally evaluate their binding relationship, we conducted a chromatin immunoprecipitation experiment followed by quantitative PCR (ChIP-qPCR) with specific primers. The enrichment of predicted promoter fragments was significantly higher after the pulldown by ETS1 antibody (Ab) in the presence of sorafenib (Fig. 3H). These data indicated that ETS1 was the key TF that directly stimulated miR-23a-3p transcription under sorafenib treatment.

### MiR-23a-3p suppression potentiated sorafenib response in HCC

**Fig. 4:**
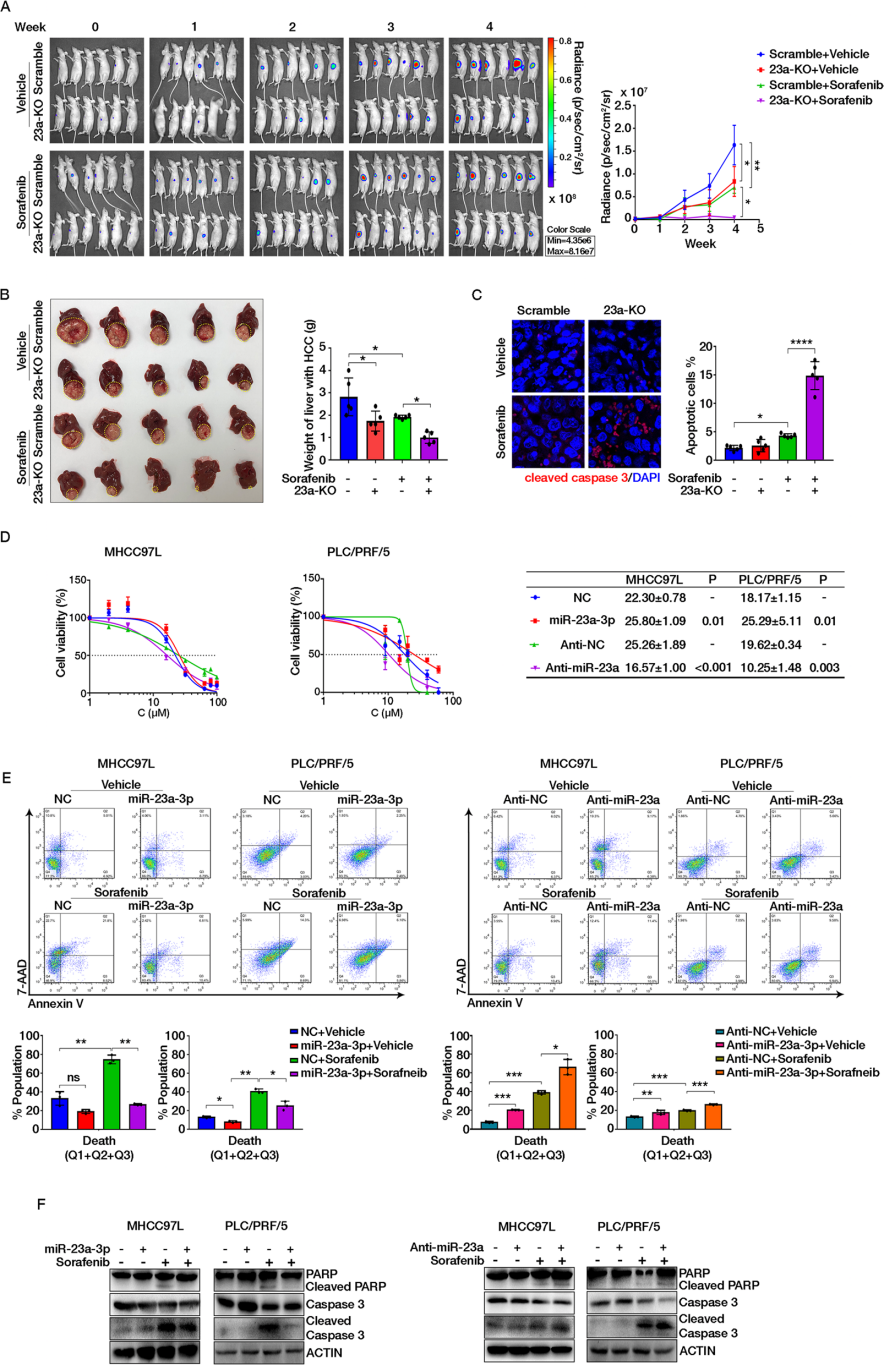
Suppression of miR-23a-3p potentiated sorafenib response both in vivo and in vitro. **A** Mice with orthotopic implantation of Scramble and 23a-KO cells (*n* = 6) and the signal intensity in HCC in 4 weeks. **B** Images and the weight of HCC-bearing livers. Yellow circles indicate HCC tumours. **C** Cleaved caspase-3 staining in the orthotopic HCC section. Cells with green staining are apoptotic cells. **D** The IC_50_ value of sorafenib after miR-23a-3p and Anti-miR-23a transfection in MHCC97L and PLC for 24 h. Mean ± SD of IC_50_ was displayed and P-value of comparison between groups was shown. **E** and **F** The effect of miR-23a-3p in cell apoptosis determined by FACS analysis using Annexin V-FITC/7-AAD double staining kit and immunoblotting

To further understand the role of miR-23a-3p in mediating sorafenib response in HCC, we established the miR-23a-3p knockout MHCC97L cell line (23a-KO) via CRISPR-Cas9 editing (Fig. S4A). The orthotopic hepatic tumour model formed by 23a-KO cells and its control pair (Scramble) was randomly grouped and received sorafenib (25 mg/kg) every other day for 4 weeks, as shown in Fig. S4B. The knockout of miR-23a-3p slightly delayed the growth of orthotopic tumours in the liver while dramatically decreased the growth of 23a-KO tumours in mice receiving sorafenib (Fig. 3A, the body weight of mice could be found in Fig. S4C). At the endpoint of the experiment, isolated livers confirmed the smaller size of tumours in 23a-KO mice than in Scramble mice administrated with sorafenib (Fig. 3B). To determine whether the inhibition of tumour growth by 23a-KO under sorafenib administration is caused by apoptosis induction in tumour cells, we stained the cleaved caspase-3 to indicate the apoptotic cell. It demonstrated that 23a-KO alone and combined with sorafenib showed significantly more apoptotic cells than their control group (Fig. 4C). The level of cleaved caspase-3 and PARP protein of 23a-KO tumours also supported the induction of apoptosis in 23a-KO cells treated with sorafenib (Fig. S4D).

In in vitro study, we transfected HCC cell lines with either miR-23a-3p mimics (miR-23a-3p) or its inhibitor (Anti-miR-23a) to up-regulate or suppress miR-23a-3p (Fig. S4E). MiR-23a-3p overexpression significantly led to the poor response of HCC cells to sorafenib, while miR-23a-3p suppression sensitized HCC to sorafenib (Fig. 4D). Such effects were independent of MEK/ERK signalling pathway (Fig. S4F). The apoptosis of HCC cells was detected by Annexin V/7-AAD and immunoblotting analysis. The results showed that miR-23a-3p overexpression inhibited cell death, which was manifested in reduced apoptotic cells and cleaved forms of caspase-3 and PARP; while miR-23a-3p suppression behaved the opposite way (Fig. 4E, F). These in vitro and in vivo observations suggested that overexpression of miR-23a-3p attenuated HCC cell response to sorafenib; and inhibition of miR-23a-3p potentiated sorafenib response.

### MiR-23a-3p overexpression attenuated sorafenib-induced ferroptosis

**Fig. 5:**
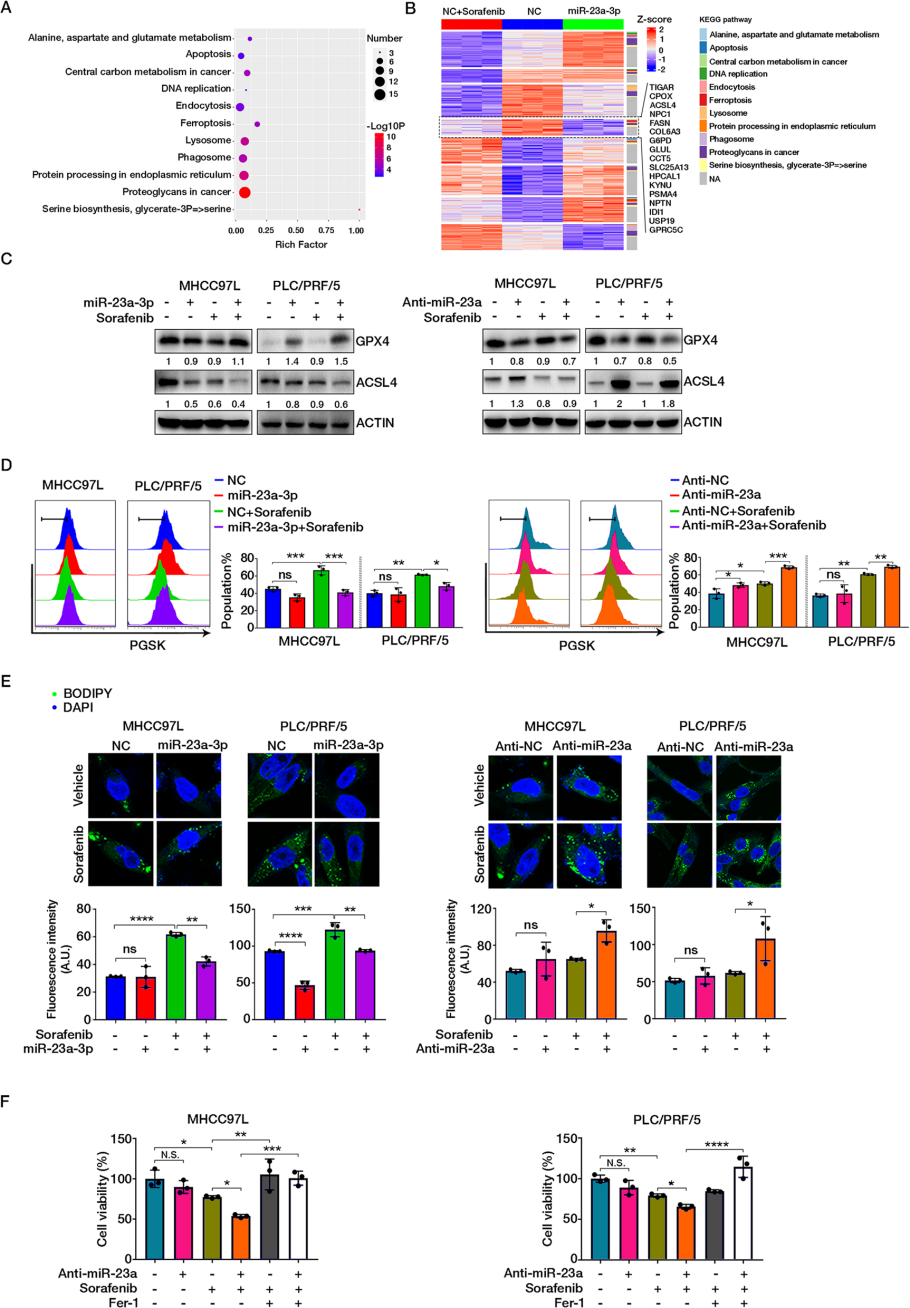
MiR-23a-3p suppressed sorafenib-induced ferroptosis. **A** Dot plot showing KEGG pathway enrichment for differentially expressed proteins among NC + sorafenib, NC, and miR-23a-3p groups. **B** Heatmap showing protein expression pattern with KEGG pathway annotation. **C** The protein expression of GPX4 and ACSL4. **D** Chelatable iron accumulation was detected by fluorescent indicator Phen Green SK with dynamic quenching signals. **E** The deposition of lipid peroxides was stained with BODIPY and measured by LSM780 confocal imaging. **F** Cell viability of HCC cells examined by MTT assay

Proteomic analysis was conducted in MHCC97L cells transfected with miR-23a-3p mimics and NC to examine potential pathways involved in miR-23a-3p-promoted sorafenib resistance. In addition, proteomics data on MHCC97L cells treated with sorafenib were also integrated for further analysis. A total of 178 differentially expressed proteins found in the three groups of cells were subjected to KEGG pathway enrichment analysis on Metascape (http://metascape.org/). Notably, molecules associated with ferroptosis were enriched together with their associated pathways such as serine biosynthesis, glutamate metabolism, apoptosis, lysosome, and protein processing in the endoplasmic reticulum (Fig. 5A).

Recent studies suggested that ferroptosis is a form of iron-dependent programmed cell death (PCD) characterized by accumulation of peroxidised lipid products, iron overload and glutathione (GSH) synthesis which contributed to sorafenib resistance in HCC [33]. Therefore, ferroptosis might be involved in miR-23a-3p-mediated sorafenib resistance. A heatmap showing the protein expression patterns among these three groups of cells were grouped into eight clusters. The KEGG pathway of each gene in the clusters was annotated (Fig. 5B). According to the commonly inhibitory effect of miRNA and upregulation of miR-23a-3p by sorafenib, genes in cluster 4 might be potentially responsible regulators of ferroptosis. Hence, we determined the expression of the key regulator glutathione peroxidase 4 (GPX4), which inhibits ferroptosis by reducing lethal lipid peroxides (LPO) and Acyl-CoA synthetase long-chain family member 4 (ACSL4), a necessary enzyme for catalysing lipid peroxidation to trigger ferroptosis. We found that sorafenib treatment alone led to a decrease of GPX4, indicating induction of ferroptosis as supported by the previous study [34]. However, miR-23a-3p overexpression could remarkably enhance GPX4 but restraint the enhancement of ACSL4 by sorafenib, suggesting suppression of sorafenib-induced ferroptosis. The opposite effects caused by Anti-miR-23a further confirmed the above result (Fig. 5C). The cellular level of excessive chelatable iron was measured by fluorescent indicator Phen Green SK, which functions as an indicator of ferroptosis initiation leading to a dynamic fluorescence quenching. A significant reduction of intracellular iron in miR-23a-3p overexpressed HCC cells was observed in the presence of sorafenib. In contrast, an augmentation of intracellular iron induced by sorafenib was seen in Anti-miR-23a HCC cells (Fig. 5D). The detection of lipid peroxides deposition by BODIPY staining also suggested that miR-23a-3p-overexpression prominently attenuate sorafenib-induced ferroptosis (Fig. 5E). Moreover, the augmented sorafenib-induced cell death by Anti-miR-23a was diminished when we used ferrostatin-1 (Fer-1), an effective ferroptosis inhibitor (Fig. 5F). Taken together, these results suggested that miR-23a-3p could suppress sorafenib-induced ferroptotic cell death in HCC cells.

### MiR-23-3p inhibited ferroptosis by targeting the 3’UTR of ACSL4

**Fig. 6:**
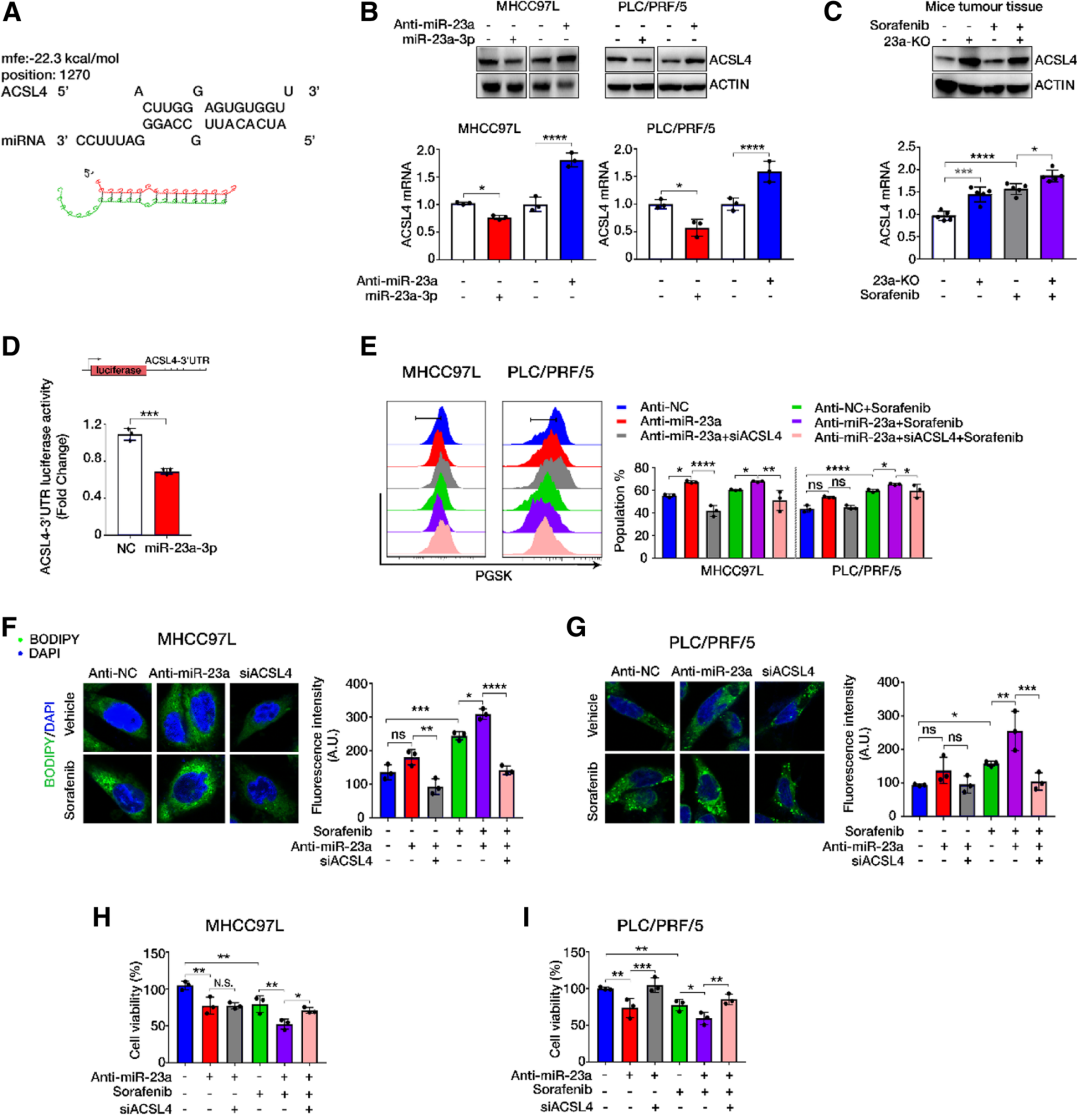
ACSL4 was targeted by miR-23a-3p. **A** Predicted miR-23a-3p binding sites in the 3’UTR of ACSL4 mRNA according to the computational algorithms of RNA hybrid. **B** The mRNA and protein expression of ACSL4 in HCC cell lines after transfection with miR-23a-3p mimics and Anti-miR-23a for 24 h. **C** ACSL4 expression in the orthotopic HCC tissues showing high ACSL4 in the 23a-KO group. **D** Luciferase activity of ACSL4 3’UTR after miR-23a-3p mimics transfection in HEK239 cells. **E** ACSL4 siRNA neutralized the induced accumulation of chelatable iron by Anti-miR-23a upon sorafenib treatment. **F** and **G** ACSL4 siRNA inhibited the deposition of lipid peroxides increased by miR-23a-3p inhibitor upon sorafenib treatment. **H** and **I** Suppression of cell viability by miR-23a-3p inhibitor under sorafenib treatment reversed by ACSL4 siRNA co-transfection

The protein expression pattern in the 4th cluster of the heatmap suggested that these proteins were associated with the effect of sorafenib treatment. Therefore, these proteins might be potential targets of miR-23a-3p as they were significantly decreased in miR-23a-3p overexpressed cells, and their expression upon sorafenib treatment was restrained by miR-23a-3p overexpression. Among these proteins, ACSL4 and CPOX (Coproporphyrinogen oxidase) were enriched in ferroptosis, which have been confirmed to be suppressed by miR-23a-3p under sorafenib treatment. Therefore, we narrowed the search scope of target genes to these two genes. The calculation by the algorithm of RNA hybrid showed a more favourable base pairing between the “seed” region of miR-23a-3p and the 3’UTR of ACSL4 mRNA compared to CPOX 3’UTR (Fig. 5A, Fig. S5A). Therefore, we selected ACSL4 for further validation. By detecting ACSL4 expression in HCC cells with miR-23a-3p mimics or Anti-miR-23a, and 23-KO HCC tumour tissues, it was found that miR-23a-3p negatively regulated the mRNA and protein expression of ACSL4 in HCC cells and tumour tissues (Fig. 6B, C). Luciferase assay demonstrated that miR-23a-3p significantly inhibited the luciferase activity of ACSL4 3’UTR (Fig. 6D), which confirmed that ACSL4 was a target gene of miR-23a-3p.

To further examine if ACSL4 was required for miR-23a-3p-suppressed ferroptosis in sorafenib-treated HCC, we co-transfected miR-23a-3p inhibitor with ACSL4 siRNA to HCC cells in the presence or absence of sorafenib (Fig. S5B, C). Suppression of ACSL4 markedly weakened the Anti-miR-23a-induced cellular iron deposition and the lipid peroxides accumulation in the presence of sorafenib (Fig. 6E-G). In addition, the induction of total reactive oxygen species (ROS) by co-treatment of sorafenib and Anti-miR-23a was also limited by ACSL4 inhibition (Fig. S5C, D). Sorafenib-induced ferroptotic cell death was determined by MTT assay. It showed that the augmentation of sorafenib-induced ferroptotic cell death by Anti-miR-23a could be attenuated by ACSL4 siRNA (Fig. 6H, I). The negative correlation between miR-23a-3p and ACSL4 was identified in human HCC TMA via dual-staining of miR-23a-3p and ACSL4 (Fig. 5E, F). These results demonstrated that ACSL4 was the target of miR-23a-3p that prominently regulated sorafenib-induced ferroptosis.

## Discussion

**Fig. 7:**
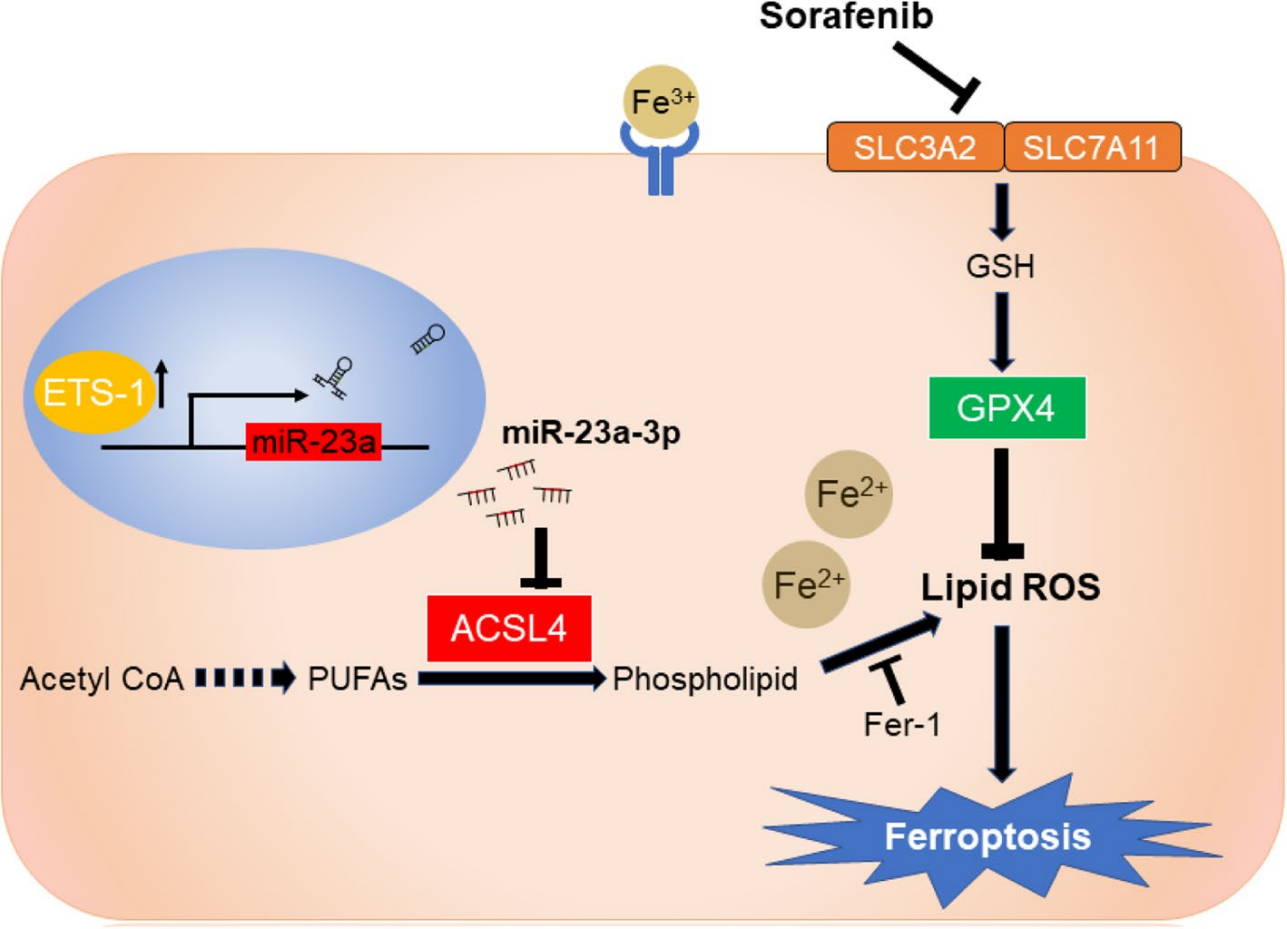
Schematic model of the mechanism underlying miR-23a-3p on sorafenib resistance in HCC. Sorafenib treatment triggered ferroptosis via lipid ROS production and chelatable iron accumulation. The ETS1 upregulated by sorafenib was a key transcription factor of miR-23a-3p that directly enhanced miR-23a-3p expression. MiR-23a-3p recognized and bound to ACSL4 3’UTR to limit lipid ROS production, thus attenuating sorafenib-induced ferroptotic cell death in HCC

Epigenetic change, especially the change of miRNAs, has been known as one of the key factors that modulate sorafenib resistance in HCC. Aberrant expression of miR-23a-3p was often observed in HCC and correlated with abnormal cell cycle, apoptosis, migration, invasion, metabolism, immune response, and more [35]. Our previous studies showed that miR-23a-3p could influence the responsiveness to topoisomerase inhibitors and mediated the activation of p53 upon DNA double-strand break in HCC cells [27, 36]. Interestingly, here we found that miR-23a-3p upregulation attenuates sorafenib-induced ferroptotic death in HCC (Fig. 7). Distinct from other forms of cell death, ferroptosis is a newly identified programmed cell death that mainly associates with iron metabolism and lipid peroxidation and participates in tumorigenesis and cancer progression in human cancers [37, 38]. It was suggested that targeting ferroptosis is a promising strategy for the treatment of some therapy-resistant tumours [39, 40]. Although the clinical significance of ferroptosis in HCC has not yet been well understood, analysis of the expression data of several ferroptosis-related genes in HCC specimens revealed that ferroptosis might predict better survival of HCC patients. Consistent with previous studies, our results demonstrated that sorafenib is a robust inducer of ferroptosis in HCC cells [41–43], and such effect may be independent of kinase inhibition [34]. Recent studies have uncovered two endogenous suppressors of sorafenib-induced ferroptotic death: metallothionein (MT)-1G and Branched-chain amino acid aminotransferase 2 (BCAT2) [44, 45]. Here, we reported a new mechanism of sorafenib resistance, suggesting that sorafenib could induce the expression of miR-23a-3p, which acts as an epigenetic suppressor against ferroptotic cell death of HCC. Our findings demonstrate a novel driving force of sorafenib resistance in HCC.

In the present study, we identified several key genes responsible for ferroptosis. GPX4 was identified as the essential negative regulator of ferroptosis, which facilitates the production of the intracellular lipid ROS, a lethal signal of cell fate. The reduction of GPX4 is considered to be a signal of ferroptosis activation. In contrast, ACSL4 was a pro-ferroptotic enzyme that catalyses the esterification of CoA to free fatty acids in an ATP-dependent manner. Specifically, long-chain polyunsaturated fatty acids (PUFAs), such as arachidonic acid (AA) and adrenic acid (AdA), are preferentially involved in lipid peroxidation. Due to its indispensable role in lipid composition, ACSL4 was an essential biomarker indicating ferroptosis [46]. According to one latest study, ACSL4 expression was relatively high in HCC patients with complete or partial response to sorafenib treatment, suggesting it could be a biomarker to predict sorafenib sensitivity in HCC [47]. However, the regulatory mechanism on ACSL4 remains a compelling question. In this study, the expression pattern of ACSL4 in proteomics data indicated that ACSL4 was responsible for miR-23a-3p mediated sorafenib resistance. Our results revealed that ACSL4 was the direct target of miR-23a-3p in mediated the suppressive effect of miR-23a-3p on ferroptosis.

It has been well known that sorafenib could trigger both apoptotic and ferroptotic cell death in HCC cells. Although ferroptosis and apoptosis are distinct forms of cell death, previous studies have suggested that crosstalk between ferroptosis and apoptosis may happen in some circumstances. Ferroptosis-inducing agents may trigger the expression of endogenous pro-apoptotic molecules such as death receptor 5 and therefore promoted cell apoptosis [48]. More importantly, it was observed that oxidative stress in ferroptotic cells due to the overload of lipid peroxides might trigger activation of mitochondria and endoplasmic reticulum-related signalling pathways that facilitate apoptotic cell death [49, 50]. Our results demonstrated that miR-23a-3p inhibition could effectively exaggerate intracellular ROS overload in sorafenib-treated HCC cells. The increased redox imbalance by miR-23a-3p inhibition may bring forward mitochondrial and ER stress, leading to enhanced apoptotic cell death. Moreover, we and others have identified a series of investigational small molecules from synthetic chemical pools and Chinese medicinal herbs [35]. It is worth exploring the possible combinations of treatments targeting miR-23a-3p in the future for a better therapeutic outcome for HCC patients.

Although the abnormal expression of miR-23a-3p is extensively observed in HCC and other cancers, only a few TFs (e.g., Runx2, c-Myc, and p53) have been proved to directly regulate the transcription of miR-23a-3p in the corresponding process. We found that ETS1, as a novel TF of miR-23a-3p, directly activated miR-23a-3p expression following sorafenib treatment. ETS1 is an ETS domain transcription family member that recognizes a conserved GGA(A/T) sequence. Previous studies revealed its downstream genes are multiple matrix metalloproteinases (MMPs), suggesting that ETS1 was an oncogene facilitating HCC metastasis and invasion [51]. Intriguingly, it was reported the binding of ETS1 to nuclear Pregnane X receptor (PXR) significantly triggered the expression of multi-drug resistance (MDR) related genes, thereby promoting sorafenib resistance of HCC [32]. Our findings also observed ETS1 positively activated by sorafenib treatment in HCC cells. The expression pattern of miR-23a-3p and its downstream target ACSL4 suggested a novel ETS-1-microRNA-mRNA regulatory network in sorafenib resistant HCC.

## Conclusion

In conclusion, we demonstrated that miR-23a-3p presents clinical significance in predicting poor response to sorafenib, poor PFS, and relapse in HCC patients. Overexpression of miR-23a-3p was observed in sorafenib-resistant HCC. Knocking out/down of miR-23a-3p could significantly improve the responsiveness of orthotopic HCC tumours and HCC cells to sorafenib treatment. The miR-23a-3p negatively regulates sorafenib-induced ferroptosis by reducing iron overload and lipid peroxidation. ACSL4 is the downstream target and ETS1 is the upstream TF of miR-23a-3p. Our study revealed a new epigenetic mechanism of sorafenib resistance and suggested that miR-23a-3p could be a promising therapeutic target for sorafenib treatment in HCC.

## Supplementary Information

**Additional file 1: Supplementary Figure 1.** (A) Sample clustering and (B) the analysis of the scale-free index for various soft-thresholding powers (β). (C) Scatterplot show the correlations between gene module membership in the blue module and gene significance for sorafenib response. (D) Overall survival analysis of the top-10 enhanced miRNAs in the blue module. Data was retrieved from Kaplan-Meier Plotter of liver cancer with default setting. (E) MiR-23a-3p expression between HCC and NAT. Unpair t-test, *P* > 0.05. (F) MiR-23a-3p expression among different grades of HCC. One-way ANOVA, P > 0.05. (G) Univariable analysis of the association between survival and clinicopathologic factors. **Supplementary Figure 2.** (A) Body weight of mice was recorded every 3 days. The arrow indicates the start of sorafenib administration. WT: vehicle group; R1–5: sorafenib-treated group. (B) Body weight of re-injected mice (*n* = 5). WT: mice with parental cells; R1/3/5: mice with in vivo-generated sorafenib resistant cells. **Supplementary Figure 3.** (A) The IC50 value of sorafenib in MHCC97L and PLC/PRF/5 by MTT assay. (B) The expression of p-ERK was downregulated after different doses of sorafenib treatment, indicating the effective response to sorafenib (C) The inhibition of ETS1 siRNAs on ETS1 mRNA and protein expression. Three biological replicates were conducted independently in all experiments above. One-way ANOVA, *P < 0.05, **P < 0.01, ***P < 0.005, ****P < 0.0001. **Supplementary Figure 4.** (A) The knockout of miR-23a-3p in 23a-KO cells was determined by qRT-PCR. (B) The flow scheme illustrates orthotopic HCC mouse model establishment. (C) The body weight of mouse model. (D) The accumulation of cleaved caspase 3 and PARP was detected by immunoblotting. Total caspase 3 and PARP were determined as reference. (E) The expression of miR-23a-3p upon transfection of miR-23a-3p mimics and Anti-miR-23a. Ten nanometer of miR-23a-3p and 30 nM of Anti-miR-23a were used in the transfection experiments. (F) The downregulated phosphorylated-ERK indicated that miR-23a-3p expression did not influence sorafenib efficiency. Three biological replicates were conducted independently in all experiments above. Unpair t-test a, ****P < 0.0001, or One-way ANOVA c and e, *P < 0.05, **P < 0.01, ***P < 0.005, ****P < 0.0001. **Supplementary Figure 5.** (A) Predicted miR-23a-3p binding sites in the 3’UTR of CPOX mRNA according to the computational algorithms of RNA hybrid. (B) The inhibitory effect on ACSL4 mRNA and protein expression by siRNA interference. (C) The ACSL4 expression on cotreatment of Anti-miR-23a and sorafenib. p-Akt was induced by cellular ROS and showed a consistent pattern with ACSL4. (D) Cellular ROS was determined by DCFDA staining. (E) Tissues in data analysis were numbered from 1 to 88, two of HCC tissues were excluded due to the severe damage. (F) Correlation between miR-23a-3p and ACSL4. Three biological replicates were conducted independently in all experiments above. One-way ANOVA, *P < 0.05, **P < 0.01, ***P < 0.005, ****P < 0.0001. **Table S1.** Patient information. **Table S2.** Sequence of primer sets.

## Data Availability

The datasets used and/or analysed during the current study are available from the corresponding author on reasonable request.

## Abbreviations

ACSL4: Acyl-CoA synthetase long-chain family member 4
ABC transporters: ATP-binding cassette transporter
CMC: Carboxymethyl cellulose
ChIP: Chromatic immunoprecipitation
ERK: Extracellular signal-regulated kinase
EMT: Epithelial-to-mesenchymal transition
ETS-1: Erythroblast Transformation Specific proto-oncogene 1
FDA: Food and Drug Administration
GEO: Gene Expression Omnibus
GSH: Glutathione
GPX4: Glutathione peroxidase 4
HCC: Hepatocellular carcinoma
HR: Hazard ratio
IC_50_: The half-maximal inhibitory concentration
MiR: MicroRNA
ME: Module eigengene
MM: Module membership
NAT: Normal adjacent tissue
OS: Overall survival
PCD: Programmed cell death
PFS: Progression-free survival
PARP: Poly (ADP-ribose) polymerase
PUFA: Polyunsaturated fatty acid
SLC: Solute carrier
SHARP: Sorafenib HCC assessment randomized protocol
TFs: Transcription factors
TMA: Tissue microarray
TAM: Tumour-associated microenvironment
TKI: Tyrosine kinase inhibitor
WGCNA: Weighted gene co-expression network analysis
