# Supplementary material for: Epigenetic regulation of ferroptosis via ETS1/miR-23a-3p/ACSL4 axis mediates sorafenib resistance in human hepatocellular carcinoma
Source: J Exp Clin Cancer Res. 2022 Jan 3;41:3. doi: 10.1186/s13046-021-02208-x (PMC8722264; doi:10.1186/s13046-021-02208-x)
Supplement: Supplementary file 1 — Additional file 1: Supplementary Figure 1. (A) Sample clustering and (B) the analysis of the scale-free index for various soft-thresholding powers (β). (C) Scatterplot show the correlations between gene module membership in the blue module and gene significance for sorafenib response. (D) Overall survival analysis of the top-10 enhanced miRNAs in the blue module. Data was retrieved from Kaplan-Meier Plotter of liver cancer with default setting. (E) MiR-23a-3p expression between HCC and NAT. Unpair t-test, P > 0.05. (F) MiR-23a-3p expression among different grades of HCC. One-way ANOVA, P > 0.05. (G) Univariable analysis of the association between survival and clinicopathologic factors. Supplementary Figure 2. (A) Body weight of mice was recorded every 3 days. The arrow indicates the start of sorafenib administration. WT: vehicle group; R1–5: sorafenib-treated group. (B) Body weight of re-injected mice (n = 5). WT: mice with parental cells; R1/3/5: mice with in vivo-generated sorafenib resistant cells. Supplementary Figure 3. (A) The IC50 value of sorafenib in MHCC97L and PLC/PRF/5 by MTT assay. (B) The expression of p-ERK was downregulated after different doses of sorafenib treatment, indicating the effective response to sorafenib (C) The inhibition of ETS1 siRNAs on ETS1 mRNA and protein expression. Three biological replicates were conducted independently in all experiments above. One-way ANOVA, *P < 0.05, **P < 0.01, ***P < 0.005, ****P < 0.0001. Supplementary Figure 4. (A) The knockout of miR-23a-3p in 23a-KO cells was determined by qRT-PCR. (B) The flow scheme illustrates orthotopic HCC mouse model establishment. (C) The body weight of mouse model. (D) The accumulation of cleaved caspase 3 and PARP was detected by immunoblotting. Total caspase 3 and PARP were determined as reference. (E) The expression of miR-23a-3p upon transfection of miR-23a-3p mimics and Anti-miR-23a. Ten nanometer of miR-23a-3p and 30 nM of Anti-miR-23a were used in the transfection experim [file 13046_2021_2208_MOESM1_ESM.docx]

**Supplementary Information**

**Epigenetic regulation of ACSL4-associated ferroptosis mediates sorafenib resistance in human hepatocellular carcinoma**

Yuanjun Lu^1^, Yau-Tuen Chan^1^, Hor-Yue Tan^2^, Cheng Zhang^1^, Wei Guo^3^, Yu Xu^1^, Rakesh Sharma^4^, Zhe-Sheng Chen^5^, Yi-Chao Zheng^6^, Ning Wang^1,*^, Yibin Feng^1,*^

1. School of Chinese Medicine, The University of Hong Kong, Pokfulam, Hong Kong.

2. Centre for Chinese Herbal Medicine Drug Development, School of Chinese Medicine, Hong Kong Baptist University, Hong Kong.

3. Department of Pharmacy, Union Hospital, Tongji Medical College, Huazhong University of Science and Technology, Hubei Province, P.R. China.

4. Proteomics and Metabolomics Core Facility, The University of Hong Kong, Pokfulam, Hong Kong, Hong Kong.

5. Department of Pharmaceutical Sciences, College of Pharmacy and Health Sciences, St. John's University, Queens, NY, United States.

6. School of Pharmaceutical Sciences, Zhengzhou University, Henan Province, P.R. China.

**Corresponding authors:** Ning Wang ([ckwang@hku.hk](mailto:ckwang@hku.hk))

Yibin Feng ([yfeng@hku.hk](mailto:yfeng@hku.hk))

**SUPPLEMENTARY FIGURE 1**


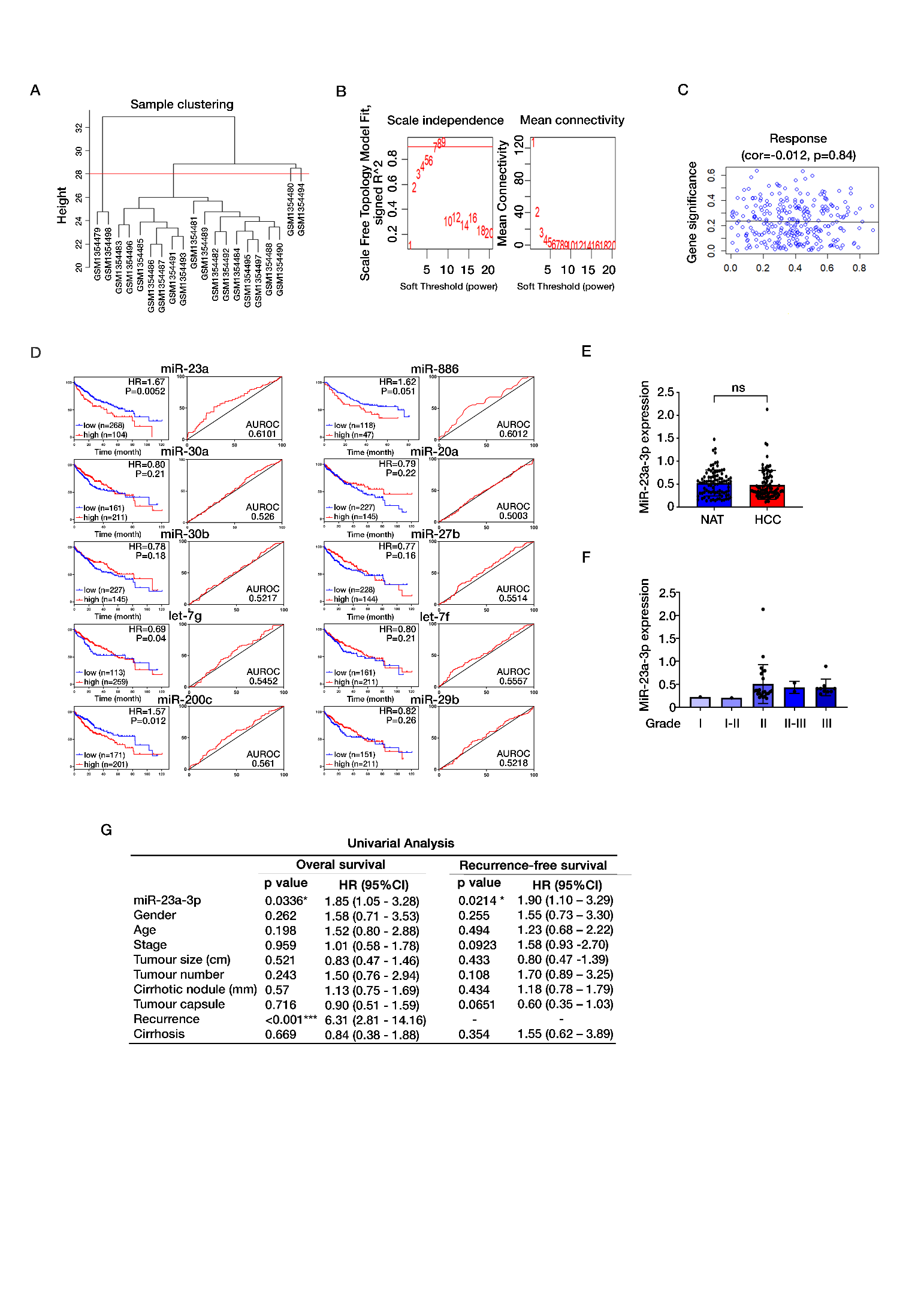


**Supplementary Figure 1.** (A) Sample clustering and (B) the analysis of the scale-free index for various soft-thresholding powers (β). (C) Scatterplot show the correlations between gene module membership in the blue module and gene significance for sorafenib response. (D) Overall survival analysis of the top-10 enhanced miRNAs in the blue module. Data was retrieved from Kaplan-Meier Plotter of liver cancer with default setting. (E) MiR-23a-3p expression between HCC and NAT. Unpair t-test, P>0.05. (F) MiR-23a-3p expression among different grades of HCC. One-way ANOVA, P>0.05. (G) Univariable analysis of the association between survival and clinicopathologic factors.

**SUPPLEMENTARY FIGURE 2**


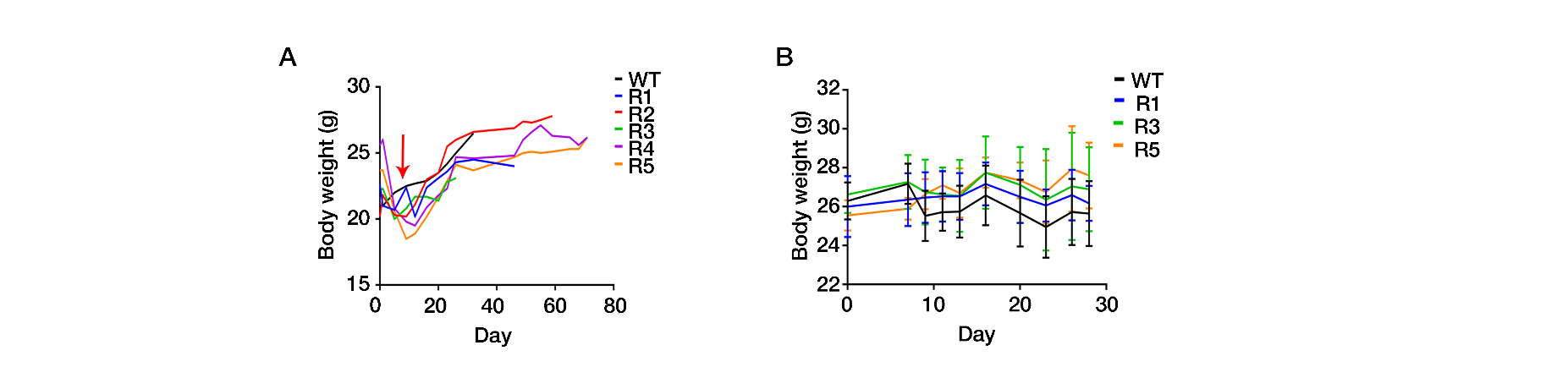


**Supplementary Figure 2.** (A) Body weight of mice was recorded every three days. The arrow indicates the start of sorafenib administration. WT: vehicle group; R1-5: sorafenib-treated group.

(B) Body weight of re-injected mice (n=5). WT: mice with parental cells; R1/3/5: mice with *in vivo*-generated sorafenib resistant cells.

**SUPPLEMENTARY FIGURE 3**


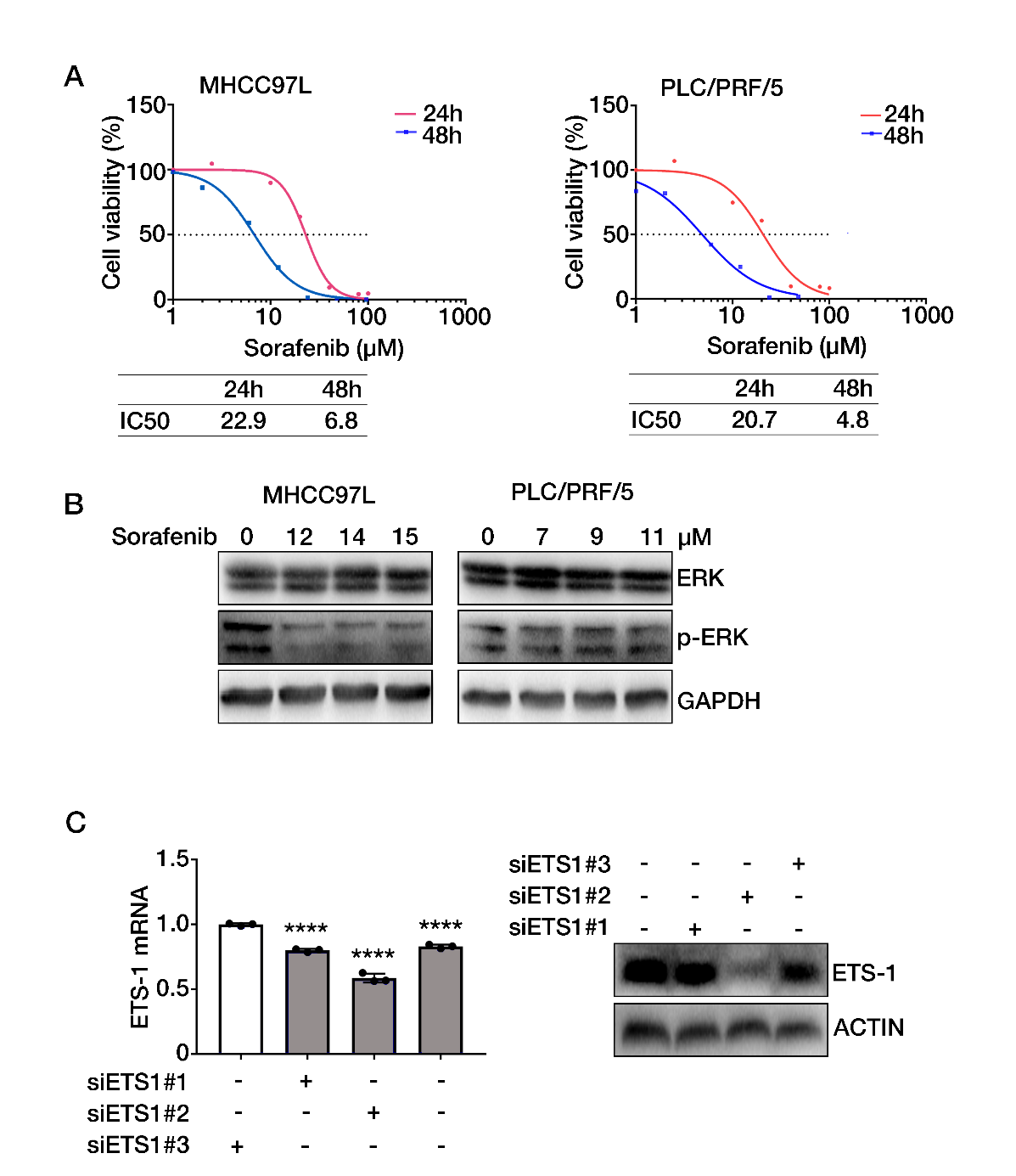


**Supplementary Figure 3.** (A) The IC50 value of sorafenib in MHCC97L and PLC/PRF/5 by MTT assay. (B) The expression of p-ERK was downregulated after different doses of sorafenib treatment, indicating the effective response to sorafenib (C) The inhibition of ETS1 siRNAs on ETS1 mRNA and protein expression. Three biological replicates were conducted independently in all experiments above. One-way ANOVA, *P<0.05, **P<0.01, ***P<0.005, ****P<0.0001.

**SUPPLEMENTARY FIGURE 4**

_
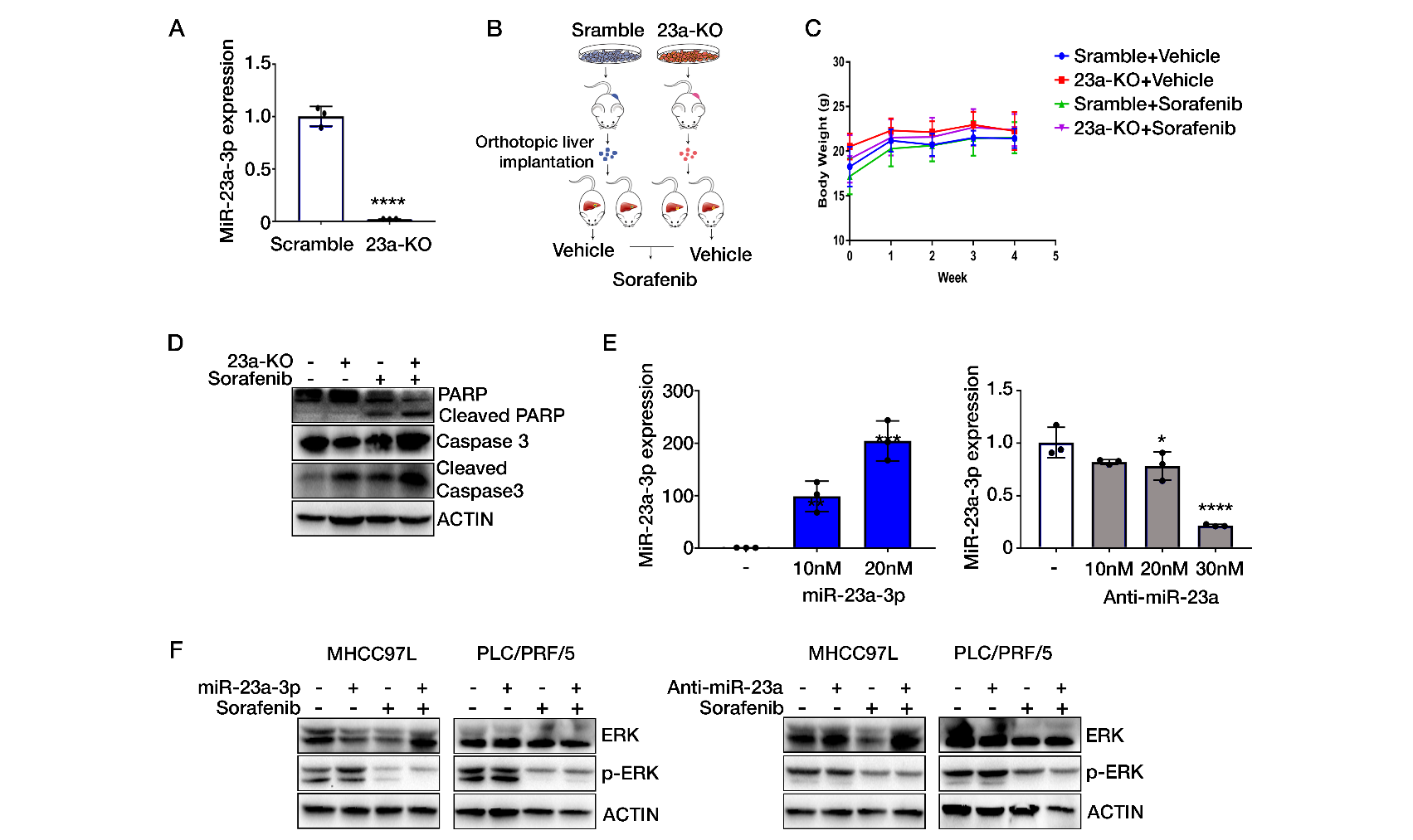
_

**Supplementary Figure 4.** (A) The knockout of miR-23a-3p in 23a-KO cells was determined by qRT-PCR. (B) The flow scheme illustrates orthotopic HCC mouse model establishment. (C) The body weight of mouse model. (D) The accumulation of cleaved caspase 3 and PARP was detected by immunoblotting. Total caspase 3 and PARP were determined as reference. (E) The expression of miR-23a-3p upon transfection of miR-23a-3p mimics and Anti-miR-23a. 10 nM of miR-23a-3p and 30 nM of Anti-miR-23a were used in the transfection experiments. (F) The downregulated phosphorylated-ERK indicated that miR-23a-3p expression did not influence sorafenib efficiency. Three biological replicates were conducted independently in all experiments above. Unpair t-test a, ****P<0.0001, or One-way ANOVA c and e, *P<0.05, **P<0.01, ***P<0.005, ****P<0.0001.

**
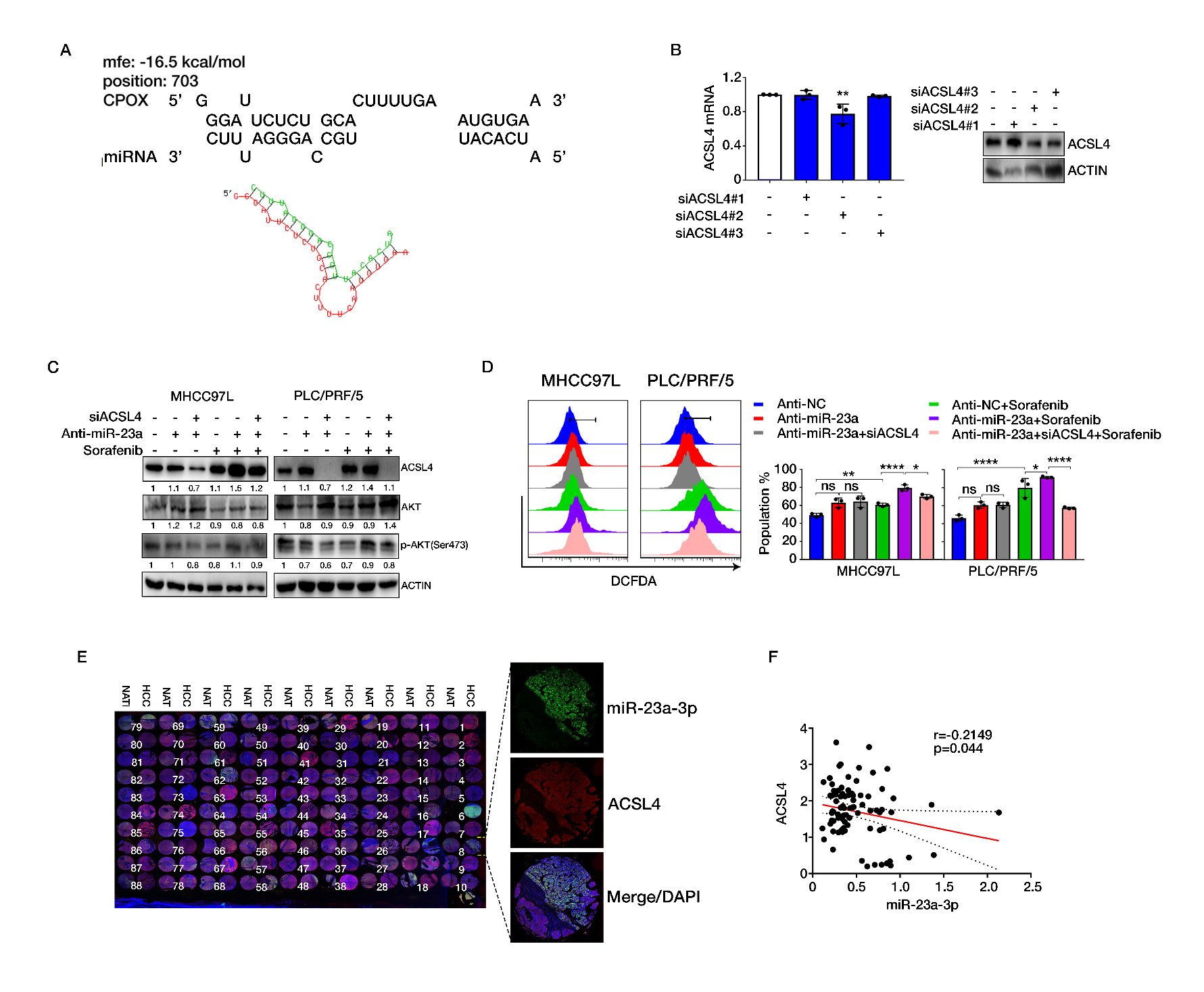
SUPPLEMENTARY FIGURE 5**

**Supplementary Figure 5.** (A) Predicted miR-23a-3p binding sites in the 3’UTR of CPOX mRNA according to the computational algorithms of RNA hybrid. (B) The inhibitory effect on ACSL4 mRNA and protein expression by siRNA interference. (C) The ACSL4 expression on cotreatment of Anti-miR-23a and sorafenib. p-Akt was induced by cellular ROS and showed a consistent pattern with ACSL4. (D) Cellular ROS was determined by DCFDA staining. (E) Tissues in data analysis were numbered from 1 to 88, two of HCC tissues were excluded due to the severe damage. (F) Correlation between miR-23a-3p and ACSL4. Three biological replicates were conducted independently in all experiments above. One-way ANOVA, *P<0.05, **P<0.01, ***P<0.005, ****P<0.0001.

**Supplementary Tables**

**Table S1.** Patient information

|  | **survival status** | **Survival months** | **Gender** | **Age** | **Stage** | **Tumour size (cm)** | **Tumour number** | **Cirrhotic nodule** | **Tumour capsule** | **Recurrence** | **Tumour-free survival months** | **Cirrhosis** |
| --- | --- | --- | --- | --- | --- | --- | --- | --- | --- | --- | --- | --- |
| 1 | Deceased | 51 | F | 73 | Ⅱ | 4 | 1 | <3mm | No | Yes | 48 | Positive |
| 2 | Survived | 72 | F | 37 | Ⅱ | 1.6 | 1 | <3mm | Yes | No | 72 | Positive |
| 3 | Deceased | 34 | M | 53 | Ⅱ | 4.5 | 3 | >3mm | No | Yes | 12 | Positive |
| 4 | Survived | 71 | M | 41 | Ⅲ | 7 | 2 | 0 | Yes | Yes | 5 | Positive |
| 5 | Deceased | 23 | F | 80 | Ⅱ | 7 | 1 | >3mm | No | Yes | 20 | Negative |
| 6 | Survived | 71 | M | 56 | Ⅱ | 1.8 | 1 | <3mm | No | No | 71 | Positive |
| 7 | Deceased | 53 | M | 41 | Ⅱ | 3 | 1 | <3mm | Yes | Yes | 15 | Positive |
| 8 | Survived | 70 | M | 52 | Ⅱ-Ⅲ | 4 | 1 | 0 | No | Yes | 9 | Positive |
| 9 | Deceased | 47 | M | 49 | Ⅱ | 4.5 | 1 | >3mm | No | Yes | 29 | Positive |
| 10 | Deceased | 19 | M | 50 | Ⅲ | 5.5 | 2 | <3mm | Yes | No | 19 | Positive |
| 11 | Survived | 69 | F | 43 | Ⅱ | 3 | 1 | <3mm | No | No | 69 | Positive |
| 12 | Deceased | 57 | M | 63 | Ⅱ | 4 | 1 | >3mm | No | Yes | 51 | Positive |
| 13 | Survived | - | M | 43 | Ⅲ | 7 | 1 | >3mm | No | Yes | 50 | Positive |
| 14 | Deceased | 19 | M | 38 | Ⅱ-Ⅲ | 7 | 1 | >3mm | No | Yes | 12 | Positive |
| 15 | Deceased | 13 | M | 45 | Ⅲ | 12 | 4 | <3mm | Yes | Yes | 4 | Positive |
| 16 | Deceased | 40 | F | 59 | Ⅱ | 8 | 1 | >3mm | Yes | Yes | 16 | Negative |
| 17 | Survived | 68 | M | 56 | Ⅱ | 8 | 1 | >3mm | Yes | No | 68 | Positive |
| 18 | Survived | 67 | M | 46 | Ⅱ | 3.5 | 1 | 0 | Yes | No | 67 | Positive |
| 19 | Deceased | 41 | M | 27 | Ⅱ | 11 | 1 | <3mm | Yes | Yes | 32 | Positive |
| 20 | Deceased | 20 | M | 54 | Ⅱ | 3.5 | 1 | >3mm | Yes | Yes | 7 | Negative |
| 21 | Survived | 65 | M | 50 | Ⅱ | 2.8 | 2 | >3mm | No | Yes | 25 | Positive |
| 22 | Survived | 65 | M | 59 | Ⅱ | 12 | 1 | >3mm | Yes | No | 65 | Positive |
| 23 | Deceased | 17 | M | 59 | Ⅱ-Ⅲ | 12 | 1 | <3mm | No | Yes | 6 | Positive |
| 24 | Survived | 65 | M | 44 | Ⅱ | 4 | 1 | >3mm | Yes | No | 65 | Positive |
| 25 | Deceased | 10 | M | 39 | Ⅱ | 5 | 1 | <3mm | No | Yes | 5 | Positive |
| 26 | Survived | 65 | F | 70 | Ⅱ | 10.5 | 1 | <3mm | Yes | No | 65 | Positive |
| 27 | Deceased | 31 | M | 51 | Ⅱ | 3 | 1 | >3mm | Yes | No | 31 | Negative |
| 28 | Deceased | 13 | M | 54 | Ⅱ | 9 | 1 | <3mm | No | Yes | 5 | Positive |
| 29 | Deceased | 7 | M | 48 | Ⅱ | 9 | 1 | <3mm | No | Yes | 5 | Positive |
| 30 | Deceased | 48 | M | 35 | Ⅱ | 2.5 | 3 | >3mm | Yes | Yes | 12 | Positive |
| 31 | Deceased | 13 | M | 53 | Ⅱ | 7 | 1 | <3mm | No | Yes | 10 | Positive |
| 32 | Deceased | 16 | M | 48 | Ⅲ | 5 | 3 | >3mm | Yes | Yes | 16 | Positive |
| 33 | Survived | 63 | M | 46 | Ⅱ | 2.5 | 1 | >3mm | Yes | Yes | 53 | Positive |
| 34 | Survived | - | M | 29 | Ⅲ | 3 | 1 | >3mm | No | No |  | Positive |
| 35 | Survived | 62 | M | 62 | Ⅱ | 5.5 | 2 | <3mm | No | No | 62 | Positive |
| 36 | Survived | 61 | M | 57 | Ⅲ | 4 | 1 | <3mm | Yes | No | 61 | Negative |
| 37 | Deceased | 15 | M | 79 | Ⅲ | 14 | 1 | 0 | No | Yes | 9 | Positive |
| 38 | Survived | 60 | M | 39 | Ⅱ | 5.5 | 2 | <3mm | No | No | 60 | Positive |
| 39 | Deceased | 22 | M | 39 | Ⅱ | 6.5 | 2 | 0 | No | Yes | 5 | Positive |
| 40 | Survived | 60 | F | 55 | Ⅰ | 8 | 1 | 0 | No | No | 60 | Positive |
| 41 | Survived | 60 | M | 59 | Ⅲ | 2.5 | 1 | >3mm | No | Yes | 45 | Positive |
| 42 | Survived | 60 | F | 60 | Ⅱ | 1 | 2 | >3mm | Yes | Yes | 56 | Positive |
| 43 | Survived | 60 | M | 59 | Ⅲ | 2 | 1 | <3mm | No | No | 60 | Negative |
| 44 | Deceased | 9 | M | 75 | Ⅲ | 3.5 | 1 | >3mm | Yes | Yes | 7 | Negative |
| 45 | Deceased | 37 | M | 66 | Ⅱ | 3.5 | 1 | >3mm | No | No | 37 | Negative |
| 46 | Survived | 60 | M | 34 | Ⅱ | 1.2 | 1 | >3mm | Yes | No | 60 | Positive |
| 47 | Deceased | 27 | M | 42 | Ⅱ | 6 | 1 | >3mm | No | Yes | 15 | Positive |
| 48 | Deceased | 13 | M | 41 | Ⅱ | 4.5 | 1 | <3mm | Yes | Yes | 7 | Positive |
| 49 | Deceased | 12 | M | 57 | Ⅱ | 8 | 1 | >3mm | Yes | No | 12 | Positive |
| 50 | Deceased | 24 | M | 59 | Ⅱ | 10 | 1 | 0 | No | Yes | 13 | Positive |
| 51 | Survived | 59 | M | 51 | Ⅱ-Ⅲ | 3.5 | 1 | >3mm | Yes | Yes | 12 | Negative |
| 52 | Deceased | 16 | M | 84 | Ⅱ | 5.5 | 1 | <3mm | No | Yes | 12 | Positive |
| 53 | Survived | 59 | M | 39 | Ⅲ | 6.5 | 1 | >3mm | No | Yes | 4 | Positive |
| 54 | Deceased | 47 | M | 60 | Ⅲ | 2.2 | 1 | <3mm | Yes | Yes | 42 | Positive |
| 55 | Survived | 59 | M | 40 | Ⅲ | 11 | 1 | >3mm | No | Yes | 7 | Positive |
| 56 | Deceased | 12 | M | 35 | Ⅲ | 2.5 | 1 | <3mm | Yes | No | 12 | Negative |
| 57 | Survived | 59 | F | 60 | Ⅲ | 6.5 | 1 | >3mm | Yes | No | 59 | Positive |
| 58 | Deceased | 44 | M | 56 | Ⅱ | 6.5 | 2 | <3mm | Yes | Yes | 37 | Positive |
| 59 | Survived | 59 | M | 50 | Ⅱ | 3 | 1 | >3mm | No | Yes | 32 | Positive |
| 60 | Deceased | 38 | M | 31 | Ⅱ | 8 | 2 | <3mm | Yes | No | 38 | Positive |
| 61 | Deceased | 29 | M | 63 | Ⅲ | 14 | 1 | <3mm | Yes | Yes | 25 | Positive |
| 62 | Survived | 59 | F | 42 | Ⅲ | 2 | 1 | <3mm | Yes | No | 59 | Positive |
| 63 | Deceased | 12 | M | 47 | Ⅱ | 4 | 1 | <3mm | Yes | No | 12 | Positive |
| 64 | Deceased | 63 | M | 58 | Ⅲ | 3 | 1 | <3mm | No | Yes | 13 | Positive |
| 66 | Survived | 58 | M | 41 | Ⅱ-Ⅲ | 3.8 | 1 | >3mm | Yes | No | 58 | Negative |
| 67 | Survived | 58 | M | 72 | Ⅱ | 7 | 1 | 0 | Yes | No | 58 | Positive |
| 68 | Deceased | 13 | M | 64 | Ⅱ | 6 | 1 | 0 | No | Yes | 8 | Positive |
| 69 | Survived | 58 | M | 49 | Ⅱ | 2 | 1 | >3mm | No | No | 58 | Negative |
| 70 | Deceased | 48 | M | 50 | Ⅱ-Ⅲ | 10 | 1 | 0 | No | Yes | 7 | Positive |
| 71 | Deceased | 39 | F | 72 | Ⅱ | 6 | 1 | <3mm | Yes | Yes | 6 | Positive |
| 72 | Survived | 58 | M | 54 | Ⅲ | 1.2 | 1 | >3mm | No | No | 58 | Positive |
| 73 | Deceased | 20 | M | 39 | Ⅲ | 10 | 1 | >3mm | No | Yes | 13 | Positive |
| 74 | Deceased | 41 | M | 48 | Ⅱ | 3 | 1 | >3mm | Yes | Yes | 38 | Positive |
| 76 | Deceased | 51 | F | 56 | Ⅲ | 5 | 1 | <3mm | Yes | Yes | 19 | Positive |
| 77 | Survived | 57 | F | 45 | Ⅱ | 2.2 | 1 | <3mm | No | No | 57 | Positive |
| 78 | Survived | 57 | M | 55 | Ⅱ | 6 | 1 | <3mm | Yes | No | 57 | Positive |
| 79 | Deceased | 17 | M | 43 | Ⅱ | 2.5 | 2 | >3mm | Yes | Yes | 10 | Positive |
| 80 | Deceased | 27 | F | 64 | Ⅱ | 6 | 1 | <3mm | Yes | Yes | 21 | Positive |
| 81 | Deceased | 39 | M | 51 | Ⅲ | 2 | 1 | >3mm | Yes | Yes | 16 | Positive |
| 82 | Deceased | 41 | M | 69 | Ⅱ | 5 | 1 | >3mm | Yes | Yes | 30 | Positive |
| 83 | Deceased | 41 | F | 62 | Ⅱ | 5.5 | 2 | >3mm | No | Yes | 21 | Positive |
| 84 | Survived | 57 | M | 45 | Ⅱ | 4 | 1 | <3mm | No | No | 57 | Positive |
| 85 | Survived | 57 | M | 58 | Ⅱ | 3 | 1 | <3mm | Yes | No | 57 | Positive |
| 86 | Survived | 57 | M | 64 | Ⅱ | 3.6 | 1 | <3mm | No | No | 57 | Positive |
| 87 | Survived | 57 | M | 42 | Ⅲ | 6 | 1 | <3mm | Yes | No | 57 | Positive |
| 88 | Deceased | 8 | M | 49 | Ⅱ | 15 | 1 | >3mm | Yes | Yes | 6 | Positive |
| 89 | Survived | 57 | M | 41 | Ⅱ-Ⅲ | 2 | 1 | 0 | Yes | No | 57 | Positive |
| 90 | Deceased | 10 | M | 55 | Ⅲ | 14 | 2 | >3mm | No | Yes | 7 | Positive |

**Table S2. Sequence of primer sets**

| **Gene** | **Forward primer (5’ to 3’)** | **Reverse primer (5’ to 3’)** |
| --- | --- | --- |
| Pri-miR-23a | TCTCATATGCAGGAGCCACCA | GCAAGTTGCTGTAGCCTCCTTG |
| GAPDH | GAGTCCACTGGCGTCTTCAC | TTCACACCCATGACGAACAT |
| Primers for ChIP-qPCR | GCCACTTCCTAGAAGCCTGG | CTGGTGGGTGTGGGCTAAG |
| ACSL4 | CATCCCTGGAGCAGATACTCT | TCACTTAGGATTTCCCTGGTCC |
| ETS1 | GATAGTTGTGATCGCCTCACC | GTCCTCTGAGTCGAAGCTGTC |
| β-actin | GAGCTACGAGCTGCCTGACG | GTAGTTTCGTGGATGCCACAG |
